# New roles of Lagrange multiplier method in generalizability theory: Inference of estimating the optimal sample size for teaching ability evaluation of college teachers

**DOI:** 10.1371/journal.pone.0307710

**Published:** 2024-10-17

**Authors:** Guangming Li

**Affiliations:** Center for Studies of Psychological Application, School of Psychology, South China Normal University, Guangzhou, China; Northwestern Polytechnical University, CHINA

## Abstract

**Background:**

Generalizability theory is widely used in psychological and educational measurement. Budget and cost are the problems that cannot be neglected in the measurement. When there is a budget constraint, the generalizability theory needs to consider how to design a measurement program with relatively high reliability and feasibility, which requires the optimal sample size to be estimated by some means. Lagrange multiplier method is a commonly used method for estimating the optimal sample size under budget constraints in generalizability theory. Unfortunately, to date, many formulas of estimating the optimal sample size for some more complex generalizability designs such as those with four facets or more facets have not been derived using the Lagrange multiplier method.

**Purpose:**

The purpose of this article is to provide a detailed step-by-step derivation of the formula of estimating the optimal sample size for three typical complex generalizability designs using the Lagrange multiplier method under budget constraints in generalizability theory, which can demonstrate the new roles of the Lagrange multiplier method.

**Method:**

This article derived the optimal sample size for teaching ability evaluation of college teachers with budget constraints in three generalizability designs such as the *(s*:*t)*×*i*, *(s*:*t)*× *(i*:*v)* and *(s*:*t)* × *(i*:*v)* ×*o* and explored their practical applications. By estimating the optimal sample size, the optimal generalizability design, which is more applicable in practice, can be compared to obtain.

**Findings:**

(1) Using the Lagrange multiplier method, the optimal sample size for students and items under budget constraints in different generalizability design can be derived. (2) For an example, based on teaching ability evaluation of college teachers in China, these designs of *(s*:*t)* ×*i*, *(s*:*t)* × *(i*:*v)* and *(s*:*t)* × *(i*:*v)* ×*o* were used to obtain the optimal sample size, which indicates the Lagrange multiplier method can been used in practice. (3) Under budget constraints, the *(s*:*t) × (i*:*v)* is the optimal generalizability design. The optimal sample size of students is 17 for each teacher and the optimal sample size of items is 4 for each dimension.

**Conclusion:**

The optimal sample size can be derived carefully using the Lagrange multiplier method under budget constraints in generalizability theory. The Lagrange multiplier method with new roles is worth recommending.

## 1 Introduction

Generalizability Theory is a modern measurement theory widely used in psychological and educational practice [1, 2]. A larger generalizability coefficient often indicates higher reliability of the test. Generalizability coefficients can be improved by increasing sample size [3]. However, the sample size is subject to budget constraints. For example, the sample size may be limited by budget factors such as manpower, material resources, and financial resources, and cannot be infinitely increased [4]. Given limited conditions, increasing large level number of facets of measurement may only result in a slight change for generalizability coefficient. Therefore, how to find the balance between budget and reliability, as well as how to obtain the optimal generalizability coefficient under overall budget constraints have become an important problem that researchers need to consider. Fortunately, this problem can be transformed into the problem of estimating optimal sample size [5]. Therefore, it is important to examine how to effectively determine the sample size considering the budget constraints.

For example, generally the more students a teacher is evaluated by, the higher reliability the evaluation has [6]. However, in the process of evaluating the teaching ability of college teachers, budget and cost need to be considered and cannot be ignored when conducting measurement research [7]. In fact, the more students evaluate teachers, the higher their costs will be. Therefore, in the evaluation process, it is necessary to consider how to design a relatively feasible and reliable measurement program under budget constraints [8]. However, the current teaching abilities evaluation of college teachers often overlook budget constraints, typically involving all most students in the process and potentially leading to unnecessary costs. The sample size of the subject population is an undeniable factor that affects the reliability of evaluation results [9]. Due to practical operational limitations, it is difficult to conduct comprehensive evaluations throughout the population. Most evaluations are conducted using sampling methods, and the sample size becomes an important factor affecting the effectiveness of evaluations.

The Lagrange multiplier method is commonly used to solve the optimization problem about estimating the optimal sample size with equality constraints by introducing the Lagrange multipliers (new scalar unknowns) [10]. Let us give the function of two variables *z* = *f*(*a*, *b*) and additional condition *φ*(*a*, *b*) = 0. In order to find the extreme point of *z* = *f*(*a*, *b*) under additional conditions, the Lagrange function *F*(*a*, *b*, *λ*) = *f*(*a*, *b*) − *λφ*(*a*, *b*) should be made. Let *F*(*a*, *b*, *λ*) for *a*, *b* and *λ* in the first partial derivation be equal to zero, that is:

$$\left\{\begin{array}{l} F\prime a=ƒ\prime x\left(a,b\right)+\lambda \varphi \prime x\left(a,b\right)=0\\ F\prime b=ƒ\prime y\left(a,b\right)+\lambda \varphi \prime y\left(a,b\right)=0\\ F\prime \lambda =\varphi \left(a,b\right)=0 \end{array}\right.$$

By solving the above equation, *a*, *b* and *λ* can be got. Thus, the obtained (*a*, *b*) is just a possible extreme point of the function z = ƒ(*a*, *b*) under additional condition *φ*(*a*, *b*) = 0. If there is only one such extreme point, it can be directly determined that the obtained (*a*, *b*) is the extreme point from the actual problem.

Its geometric significance is to assume that the given objective function is *f*(*a*, *b*) with the constraint condition *φ*(*a*, *b*) = 0. The curve *L* is the family of contour lines whose constraint condition is *φ*(*a*, *b*) = 0 and objective function is *f*(*a*, *b*) = *C*. Under the condition that the partial derivative of *f*(*a*, *b*) and *φ*(*a*, *b*) is continuous, the possible extreme point of the objective function *f*(*a*, *b*) under constraint conditions *φ*(*a*, *b*) = 0 is *M*(*a*_*0*_, *b*_*0*_).

Because two curves must have a common normal line at the tangent point, the contour line of objective function at point *M*(*a*_*0*_, *b*_*0*_) has a normal vector $\left\{f_{a}^{\prime }(a_{0},b_{0}),f_{b}^{\prime }(a_{0},b_{0})\right\}$, and the curve of constraint condition at point *M*(*a*_*0*_, *b*_*0*_) has a normal vector $\left\{\varphi_{a}^{\prime }(a_{0},b_{0}),\varphi_{b}^{\prime }(a_{0},b_{0})\right\}$. The $\left\{f_{a}^{\prime }(a_{0},b_{0}),f_{b}^{\prime }(a_{0},b_{0})\right\}$ and $\left\{\varphi_{a}^{\prime }(a_{0},b_{0}),\varphi_{b}^{\prime }(a_{0},b_{0})\right\}$ are parallel, i.e. $\frac{f_{a}^{\prime }(a_{0},b_{0})}{\varphi_{a}^{\prime }(a_{0},b_{0})}=\frac{f_{b}^{\prime }(a_{0},b_{0})}{\varphi_{b}^{\prime }(a_{0},b_{0})}$. That is to say, there are real number λ which makes the following equation hold true: $\{f_{a}^{\prime }\left(a_{0},b_{0}\right),f_{b}^{\prime }\left(a_{0},b_{0}\right)\}+\lambda \{\varphi_{a}^{\prime }\left(a_{0},b_{0}\right),\varphi_{b}^{\prime }\left(a_{0},b_{0}\right)\}=0$.

It should be noted that, the tangent point between the contour line of objective function and the curve of constraint condition may not necessarily be extreme point of the objective function *f*(*a*, *b*) under constraint condition *φ*(*a*, *b*) = 0.

According to the Lagrange multiplier method, the unified formula of Lagrange function can be formulated as follows [11]:

L(x,y,λ)=f(x,y)−λφ(x,y) (1)

For example, according to Eq (1), if *s* is used as evaluation student, *i* is used as the evaluation items, *v* is used as the dimensions of evaluation items, and *o* is used as the evaluation occasions (or number of times), the Lagrange function can be expressed as [12]:

$$L\left(n_{s},n_{i},n_{v},n_{o},\lambda \right)=\sigma^{2}\left(\delta \right)-\lambda \left(cn_{s}n_{i}n_{v}n_{o}-B\right)$$ (2)

Where *n*_*s*_ represents the number of evaluation students, *n*_*i*_ is the number of evaluation items, *n*_*v*_ is the number of the dimensions of evaluation items, and *n*_*o*_ is the number of evaluation occasions. According to Eq (2), the optimal sample size of all designs can be estimated.

The Lagrange multiplier method proposed by Marcoulide and Goldstein [13] provides a new perspective for further solving the problem of estimating the optimal sample size in multidimensional design under budget constraints. Later, some scholars extended the Lagrange multiplier method to the more complex situation [7, 14, 15]. However, the research of these scholars is to divide and conquer the Lagrange multiplier method based on different generalizability designs. The focus of research is mostly on simple crossed design instead of expanding the research focus to more complex mixed design (mixed design includes both crossed design and nested design). However, in fact there are a number of generalizability designs that belong to multi-faceted mixed design. This has led to some shortcomings in current research when discussing how to estimate the optimal sample size under budget constraints. Based on existing literature, estimating the optimal sample size under budget constraints in generalizability theory can be improved from the following two aspects.

On the one hand, so far, the unified formula of Lagrange multiplier method has not yet been derived. Estimating the optimal sample size under budget constraints in generalizability theory is affected by factors such as the rounding of the total budget, the negative variance components, the fixed facet, the unbalanced design, and the optimal generalizability design [12]. In some generalizability designs, the estimated variance component may be negative, which may be due to issues with the data itself or may be some problems with parameter estimation methods, such as maximum likelihood estimation [16]. Generally, generalizability design is a design with random facets. However, in some cases, if only a certain number of facet levels are required, or if certain facet levels are immutable, this design becomes a design with fixed facets. At present, most studies have not further systematically summarized the basic principles of Lagrange multiplier method, but still based on the "divide and conquer" of different generalizability designs, nor has a relatively unified formula of Lagrange multiplier method been proposed to overcome the problems caused by these factors [7, 14, 15, 17–19]. Therefore, it is necessary to build the unified formula of Lagrange multiplier method and derive the equations if the optimal sample size for different generalizability designs, which can be remedied by subsequent research.

On the other hand, the Lagrange multiplier method is not applied to the more complex designs of generalizability theory. On the basis of previous studies, Meyer et al. [7] applied the Lagrange multiplier method previously proposed by Marcoulides and Goldstein [13, 20, 21] to estimate the optimal sample size for some generalizability designs under budget constraints, which is of great significance and shows that the Lagrange multiplier method has wide applicability. However, there are two issues for the generalizability design of Meyer et al. [7]: (1) there is no detailed derivation process for estimating the optimal sample size in different generalizability designs; (2) estimating the optimal sample size for the generalizability design of four facets or more facets has never been discussed. Comparing with previous similar studies, Meyer et al. [7] did not apply the Lagrange multiplier method to the more complex generalizability designs. Specifically, there has been little discussion on the some complex generalizability design with four facets or more facets [22–24], which may become one of the hot topics that can be further explored in depth in the future.

The purpose of this paper is to derive the equations of estimating optimal sample size under budget constraints for some more complex generalizability designs by using the unified formula of Lagrange multiplier method in generalizability theory, which can provide reference for subsequent practical applications.

## 2 Lagrange multiplier method

### 2.1 The Lagrange multiplier method of the *(s*:*t)* ×*i* design

Often, college students are required to evaluate the teaching ability of teachers on the same items, but the students taught by each teacher may be different. Therefore, this design is *(s*:*t)* ×*i*. Among them, *t* represents the teachers, *i* represents the items, *s* represents the students. The mean error variance and relative error variance of the *(s*:*t)* ×*i* design are [4]:

$$\sigma_{\bar{X}}^{2}=\frac{\sigma_{t}^{2}}{n_{t}}+\frac{\sigma_{i}^{2}}{n_{i}}+\frac{\sigma_{s:t}^{2}}{n_{s}}+\frac{\sigma_{ti}^{2}}{n_{i}}+\frac{\sigma_{si:t}^{2}}{n_{s}n_{i}}$$ (3)

$$\sigma^{2}\left(\delta \right)=\frac{\sigma_{s:t}^{2}}{n_{s}}+\frac{\sigma_{ti}^{2}}{n_{i}}+\frac{\sigma_{si:t}^{2}}{n_{s}n_{i}}$$ (4)

In Eq (3), $\sigma_{\bar{X}}^{2}$ represents the mean error variance; $\sigma_{t}^{2}$, $\sigma_{i}^{2}$, $\sigma_{s:t}^{2}$, $\sigma_{ti}^{2}$, $\sigma_{si:t,e}^{2}$ are the variance components of the teachers; the variance component of the items; the variance component of the students nested in the teachers; the variance component of the teacher-item interaction; and the variance component of the students crossover the items, but nested in the teachers with unmeasured and random sources of variation respectively. In this paper, the symbol *e* was used to denote unmeasured and random sources of variation [25]. The *n*_*t*_, *n*_*i*_ and *n*_*s*_ represent the sample size of the teachers, the sample size of the items, and the sample size of the students respectively. In Eq (4), *σ*^2^(*δ*) represents the relative error variance, and the meanings of other representation symbols are the same as in Eq (3).

The optimal number of students and items can be obtained by conducting min *F*(*n*_*s*_, *n*_*i*_, *λ*) = *σ*^2^(*δ*) − *λ*(*cn*_*s*_*n*_*i*_ − *B*) using the unified formula of Lagrange multiplier method of Eq (2) with Eqs (3) and (4). The specific process is as follows:

$$\sigma^{2}\left(\delta \right)=\frac{\sigma_{s:t}^{2}}{n_{s}}+\frac{\sigma_{ti}^{2}}{n_{i}}+\frac{\sigma_{si:t,e}^{2}}{n_{s}n_{i}},cn_{s}n_{i}-B$$

$$\begin{array}{l} \text{min}\;F\left(n_{s},n_{i},\lambda \right)=\sigma^{2}\left(\delta \right)-\lambda \left(cn_{s}n_{i}-B\right)\\ =\frac{\sigma_{s:t}^{2}}{n_{s}}+\frac{\sigma_{\text{t}i}^{2}}{n_{i}}+\frac{\sigma_{si:t,e}^{2}}{n_{s}n_{\text{i}}}-\lambda cn_{s}n_{i}+\lambda B \end{array}$$

$$\frac{\partial F}{\partial n_{s}}=-\frac{\sigma_{s:t}^{2}}{n_{s}^{2}}-\frac{\sigma_{si:t,e}^{2}}{n_{s}^{2}n_{i}}-\lambda cn_{i}$$ (5)

$$\frac{\partial F}{\partial n_{i}}=-\frac{\sigma_{ti}^{2}}{n_{i}^{2}}-\frac{\sigma_{si:t,e}^{2}}{n_{s}n_{i}^{2}}-\lambda cn_{s}$$ (6)

$$\frac{\partial F}{\partial \lambda }=-cn_{i}n_{s}+B$$ (7)

Let $\frac{\partial F}{\partial_{n_{s}}}$, $\frac{\partial F}{\partial_{n_{i}}}$, $\frac{\partial F}{\partial \lambda }$ are equal to 0:

Let (5) = 0, and get:

$$\lambda =\frac{{-n}_{i}\sigma_{s:t}^{2}-\sigma_{si:t,e}^{2}}{n_{s}^{2}n_{i}^{2}c}$$ (8)

Let (6) = 0, and get:

$$\lambda =\frac{{-n}_{s}\sigma_{ti}^{2}-\sigma_{si:t,e}^{2}}{n_{s}^{2}n_{i}^{2}c}$$ (9)

Let (7) = 0, and get:

$$B=cn_{i}n_{s},\;\frac{B}{c}=n_{i}n_{s}$$ (10)

Let (8) = (9), eliminate *λ*, and get:

$$\frac{n_{s}}{n_{i}}=\frac{\sigma_{s:t}^{2}}{\sigma_{ti}^{2}}$$ (11)

Jointly established by (10) and (11):

$$\left\{\begin{array}{l} \frac{B}{c}=n_{i}n_{s}\\ \frac{n_{s}}{n_{i}}=\frac{\sigma_{s:t}^{2}}{\sigma_{ti}^{2}} \end{array}\right.$$

Results are showed:

$$n_{s}=\sqrt{\frac{\sigma_{s:t}^{2}}{\sigma_{ti}^{2}}\frac{B}{c}}$$ (12)

$$n_{i}=\sqrt{\frac{\sigma_{ti}^{2}}{\sigma_{s:t}^{2}}\frac{B}{c}}$$ (13)

In Eq (12), *n*_*s*_ denotes the optimal number of students for *(s*:*t)* × *i* design. In Eq (13), *n*_*i*_ represents the optimal number of items for *(s*:*t)* ×*i* design.

### 2.2 The Lagrange multiplier method of the *(s*:*t)* × *(i*:*v)* design

Compared with the *(s*:*t)* × *i* design, the *(s*:*t)* × *(i*:*v)* design adds some dimensions of evaluation items such as teaching methods, teaching content, teaching attitudes, teaching organizations, and teaching effects, etc. The items in each dimension are different. The *v* represents the dimensions of evaluation items. The mean error variance and relative error variance of the *(s*:*t)* × *(i*:*v)* design [4] are:

$$\sigma_{\bar{X}}^{2}=\frac{\sigma_{t}^{2}}{n_{t}}+\frac{\sigma_{v}^{2}}{n_{v}}+\frac{\sigma_{i:v}^{2}}{n_{i}n_{v}}+\frac{\sigma_{s:t}^{2}}{n_{s}}+\frac{\sigma_{sv:t}^{2}}{n_{s}n_{v}}+\frac{\sigma_{tv}^{2}}{n_{v}}+\frac{\sigma_{ti:v}^{2}}{n_{i}n_{v}}+\frac{\sigma_{si:tv}^{2}}{n_{s}n_{i}n_{v}}$$ (14)

$$\sigma^{2}\left(\delta \right)=\frac{\sigma_{s:t}^{2}}{n_{s}}+\frac{\sigma_{sv:t}^{2}}{n_{s}n_{v}}+\frac{\sigma_{tv}^{2}}{n_{v}}+\frac{\sigma_{ti:v}^{2}}{n_{i}n_{v}}+\frac{\sigma_{si:tv}^{2}}{n_{s}n_{i}n_{v}}$$ (15)

In Eq (14), $\sigma_{\bar{X}}^{2}$ represents the mean error variance; $\sigma_{t}^{2}$, $\sigma_{v}^{2}$, $\sigma_{i:v}^{2}$, $\sigma_{s:t}^{2}$, $\sigma_{sv:t}^{2}$, $\sigma_{tv}^{2}$, $\sigma_{ti:v}^{2}$, $\sigma_{si:tv,e}^{2}$ are the variance components of the teachers; the variance component of the dimensions; the variance component of the items nested in the dimensions; the variance component of the students nested in the teachers; the variance component of the students crossover the dimensions, but nested in the teachers; the variance component of the teacher-dimension interaction; the variance component of the teachers crossover the items, but nested in the dimensions; and the variance component of the students crossover the items, but nested in the teachers and dimensions with unmeasured and random sources of variation respectively. The *n*_*t*_, *n*_*i*_, *n*_*s*_ and *n*_*v*_ represent the sample size of the teachers, the sample size of the items, the sample size of the students, and the sample size of the dimensions respectively. In Eq (15), *σ*^2^(*δ*) represents the relative error variance, and the meanings of other representation symbols are the same as in Eq (14).

The optimal number of students and items of the *(s*:*t)* × *(i*:*v)* design can be obtained by conducting min *F*(*n*_*s*_, *n*_*i*_, *n*_*v*_, *λ*) = *σ*^2^(*δ*) − *λ*(*cn*_*s*_*n*_*i*_*n*_*v*_ − *B*) using the unified formula of Lagrange multiplier method of Eq (2) with Eqs (14) and (15).


$$\sigma^{2}\left(\delta \right)=\frac{\sigma_{s:t}^{2}}{n_{s}}+\frac{\sigma_{sv:t}^{2}}{n_{s}n_{v}}+\frac{\sigma_{tv}^{2}}{n_{v}}+\frac{\sigma_{\text{ti}:v}^{2}}{n_{i}n_{v}}+\frac{\sigma_{si:tv,e}^{2}}{n_{s}n_{i}n_{v}},cn_{s}n_{i}n_{\text{v}}-B$$

$$\begin{array}{l} \text{min}\;F\left(n_{s}\text{,}n_{i},n_{v},\lambda \right)=\sigma^{2}\left(\delta \right)-\lambda \left(cn_{s}n_{i}n_{v}-B\right)\\ =\frac{\sigma_{s:t}^{2}}{n_{s}}+\frac{\sigma_{sv:t}^{2}}{n_{s}n_{v}}+\frac{\sigma_{tv}^{2}}{n_{v}}+\frac{\sigma_{\text{ti}:v}^{2}}{n_{i}n_{v}}+\frac{\sigma_{si:tv,e}^{2}}{n_{s}n_{i}n_{v}}-\lambda cn_{s}n_{i}n_{v}+\lambda B \end{array}$$

$$\frac{\partial F}{\partial n_{s}}=-\frac{\sigma_{s:t}^{2}}{n_{s}^{2}}-\frac{\sigma_{sv:t}^{2}}{n_{s}^{2}n_{v}}-\frac{\sigma_{si:tv,e}^{2}}{n_{s}^{2}{n_{i}n}_{v}}-\lambda cn_{i}n_{v}$$ (16)

$$\frac{\partial F}{\partial n_{i}}=-\frac{\sigma_{ti:v}^{2}}{n_{i}^{2}n_{v}}-\frac{\sigma_{si:tv,e}^{2}}{n_{i}^{2}n_{s}n_{v}}-\lambda cn_{s}n_{v}$$ (17)

$$\frac{\partial F}{\partial \lambda }=-cn_{i}n_{s}n_{v}+B$$ (18)

Let $\frac{\partial F}{\partial_{n_{s}}}$, $\frac{\partial F}{\partial_{n_{i}}}$, $\frac{\partial F}{\partial \lambda }$ are equal to 0:

Let (16) = 0:

$$\lambda cn_{i}n_{v}=-\frac{\sigma_{s:t}^{2}}{n_{s}^{2}}-\frac{\sigma_{sv:t}^{2}}{n_{s}^{2}n_{v}}-\frac{\sigma_{si:tv,e}^{2}}{n_{s}^{2}n_{i}n_{v}}$$

$$=\frac{{-n_{i}n_{v}\sigma }_{s:t}^{2}{-n_{i}\sigma }_{sv:t}^{2}{-\sigma }_{si:tv,e}^{2}}{n_{i}^{2}n_{i}n_{s}}$$

and get:

$$\lambda cn_{s}^{2}n_{i}^{2}n_{v}^{2}=-n_{i}n_{v}\sigma_{s:t}^{2}-n_{i}\sigma_{sv:t}^{2}-\sigma_{si:tv,e}^{2}$$ (19)

Let (17) = 0:

$$\lambda cn_{s}n_{v}=-\frac{\sigma_{ti:v}^{2}}{n_{i}^{2}n_{v}}-\frac{\sigma_{si:tv,e}^{2}}{n_{i}^{2}n_{s}n_{v}}$$

$$=\frac{{-n_{s}\sigma }_{ti:v}^{2}{-\sigma }_{si:tv,e}^{2}}{n_{i}^{2}n_{v}n_{s}}$$

and get:

$$\lambda cn_{s}^{2}n_{i}^{2}n_{v}^{2}=-n_{s}\sigma_{ti:v}^{2}-\sigma_{si:tv,e}^{2}$$ (20)

Let (18) = 0, and get:

$$B=cn_{i}n_{s}n_{v},\frac{B}{c}=n_{i}n_{s}n_{v}$$ (21)

Let (19) = (20), eliminate *λ*, and get:

$$n_{i}n_{v}\sigma_{s:t}^{2}+n_{i}\sigma_{sv:t}^{2}+\sigma_{si:tv,e}^{2}=n_{s}\sigma_{ti:v}^{2}+\sigma_{si:tv,e}^{2}$$

$$n_{i}n_{v}\sigma_{s:t}^{2}+n_{i}\sigma_{sv:t}^{2}=n_{s}\sigma_{ti:v}^{2}$$ (22)

Jointly established by (21) and (22):

$$\left\{\begin{array}{l} \frac{B}{c}=n_{i}n_{s}n_{v}\\ n_{i}n_{v}\sigma_{s:t}^{2}+n_{i}\sigma_{sv:t}^{2}=n_{s}\sigma_{ti:v}^{2} \end{array}\right.$$

Results are showed:

$$n_{s}=\sqrt{\frac{\left(n_{v}\sigma_{s:t}^{2}+\sigma_{sv:t}^{2}\right)}{n_{v}\sigma_{ti:v}^{2}}\frac{B}{c}}$$ (23)

$$n_{i}=\sqrt{\frac{\sigma_{ti:v}^{2}}{n_{v}\left(n_{v}\sigma_{s:t}^{2}+\sigma_{sv:t}^{2}\right)}\frac{B}{c}}$$ (24)

In Eq (23), *n*_*s*_ denotes the optimal number of students for *(s*:*t)* × *(i*:*v)* design. In Eq (24), *n*_*i*_ represents the optimal number of items for *(s*:*t)* × *(i*:*v)* design.

### 2.3 The Lagrange multiplier method of the *(s*:*t)* × *(i*:*v)* ×*o* design

Compared with the *(s*:*t)* × *(i*:*v)* design, the *(s*:*t)* × *(i*:*v)* ×*o* design indicates an increase in the number of evaluations, such as twice a year, so the occasions are two, *o* represents the occasions. The mean error variance and relative error variance of the *(s*:*t)* × *(i*:*v)* ×*o* design [4] are:

$$\sigma_{\bar{X}}^{2}=\frac{\sigma_{o}^{2}}{n_{o}}+\frac{\sigma_{t}^{2}}{n_{t}}+\frac{\sigma_{v}^{2}}{n_{v}}+\frac{\sigma_{s:t}^{2}}{n_{s}}+\frac{\sigma_{i:v}^{2}}{n_{i}n_{v}}+\frac{\sigma_{\text{o}t}^{\text{2}}}{n_{\text{o}}}+\frac{\sigma_{os:t}^{2}}{n_{s}n_{o}}+\frac{\sigma_{ov}^{\text{2}}}{n_{v}n_{\text{o}}}+\frac{\sigma_{oi:v}^{\text{2}}}{n_{i}n_{v}n_{\text{o}}}+\frac{\sigma_{tv}^{2}}{n_{v}}+\frac{\sigma_{ti:v}^{2}}{n_{i}n_{v}}+\frac{\sigma_{sv:t}^{2}}{n_{s}n_{v}}+\frac{\sigma_{si:tv}^{2}}{n_{s}n_{i}n_{v}}+\frac{\sigma_{otv}^{2}}{n_{v}n_{o}}+\frac{\sigma_{oti:v}^{2}}{n_{i}n_{v}n_{o}}+\frac{\sigma_{osv:t}^{2}}{n_{s}n_{v}n_{o}}+\frac{\sigma_{osi:tv}^{2}}{n_{s}n_{i}n_{v}n_{o}}$$ (25)

$$\sigma^{2}\left(\delta \right)=\frac{\sigma_{s:t}^{2}}{n_{s}}+\frac{\sigma_{ot}^{\text{2}}}{n_{o}}+\frac{\sigma_{os:t}^{2}}{n_{s}n_{o}}+\frac{\sigma_{tv}^{2}}{n_{v}}+\frac{\sigma_{ti:v}^{2}}{n_{i}n_{v}}+\frac{\sigma_{sv:t}^{2}}{n_{s}n_{v}}+\frac{\sigma_{si:tv}^{2}}{n_{s}n_{i}n_{v}}+\frac{\sigma_{otv}^{2}}{n_{v}n_{o}}+\frac{\sigma_{oti:v}^{2}}{n_{i}n_{v}n_{o}}+\frac{\sigma_{osv:t}^{2}}{n_{s}n_{v}n_{o}}+\frac{\sigma_{osi:tv}^{2}}{n_{s}n_{i}n_{v}n_{o}}$$ (26)

In Eq (25), $\sigma_{\bar{X}}^{2}$ means the mean error variance; $\sigma_{o}^{2}$, $\sigma_{t}^{2}$, $\sigma_{v}^{2}$, $\sigma_{s:t}^{2}$, $\sigma_{i:v}^{2}$, $\sigma_{ot}^{2}$,$\sigma_{os:t}^{2}$, $\sigma_{ov}^{\text{2}}$, $\sigma_{oi:v}^{\text{2}}$, $\sigma_{tv}^{2}$, $\sigma_{ti:v}^{2}$, $\sigma_{sv:t}^{2}$, $\sigma_{si:tv}^{2}$, $\sigma_{otv}^{2}$, $\sigma_{oti:v}^{2}$, $\sigma_{osv:t}^{2}$, $\sigma_{osi:tv,e}^{2}$ represent the variance component of the occasions; the variance component of the teachers; the variance component of the dimensions; the variance component of the students nested in the teachers; the variance component of the items nested in the dimensions; the variance component of the interaction between the teachers and occasions; the variance component of the interaction between the occasions and students but nested in the teachers; the variance component of the interaction between the occasions and dimensions; the variance component of the interaction between the occasions and items, but nested in the dimensions; the variance component of the interaction between the teachers and dimensions; the variance component of the interaction between the teachers and items, but nested in the dimensions; the variance component of the students crossover the dimensions, but nested in the teachers; the variance component of the interaction between the students and the items, but nested in the teachers crossover the dimensions; the variance component of the interaction of occasion-teacher-dimension; the variance component of the interaction of occasion-teacher-item, but nested in the dimensions; the variance component of the interaction of occasion-student-dimension, but nested in the teachers crossover the dimensions with unmeasured and random sources of variation respectively. The *n*_*t*_, *n*_*i*_, *n*_*s*_, *n*_*v*_ and *n*_*o*_ are the sample size of teachers, the sample size of items, the sample size of students, the sample size of dimensions, and the sample size of occasions respectively. In Eq (26), *σ*^2^(*δ*) represents the relative error variance, and the meanings of other representation symbols are the same as in Eq (25).

The optimal number of students and items can be obtained by conducting min *F*(*n*_*s*_, *n*_*i*_, *n*_*v*_, *n*_*o*_, *λ*) = *σ*^2^(*δ*) − *λ*(*cn*_*s*_*n*_*i*_*n*_*v*_*n*_*o*_ − *B*) using the unified formula of Lagrange multiplier method of Eq (2) with Eqs (25) and (26).


$$\begin{array}{l} \sigma^{2}\left(\delta \right)=\frac{\sigma_{s:\text{t}}^{2}}{n_{s}}+\frac{\sigma_{\text{o}t}^{\text{2}}}{n_{\text{o}}}+\frac{\sigma_{os:t}^{2}}{n_{o}n_{s}}+\frac{\sigma_{tv}^{2}}{n_{v}}+\frac{\sigma_{ti:v}^{2}}{n_{i}n_{v}}+\frac{\sigma_{sv:t}^{2}}{n_{s}n_{v}}+\frac{\sigma_{si:tv}^{2}}{n_{s}n_{i}n_{v}}+\frac{\sigma_{otv}^{2}}{n_{o}n_{v}}+\frac{\sigma_{oti:v}^{2}}{n_{o}n_{i}n_{v}}+\frac{\sigma_{osv:t}^{2}}{n_{o}n_{s}n_{v}}+\frac{\sigma_{osi:tv,e}^{2}}{n_{o}n_{s}n_{i}n_{v}},\\ cn_{s}n_{i}n_{\text{v}}n_{o}-B \end{array}$$

$$\begin{array}{l} \text{min}\;F\left(n_{\text{s}}\text{,}n_{i},n_{\text{v}},n_{o},\lambda \right)=\sigma^{2}\left(\delta \right)-\lambda \left(cn_{s}n_{i}n_{\text{v}}n_{o}-B\right)\\ =\frac{\sigma_{s:\text{t}}^{2}}{n_{s}}+\frac{\sigma_{\text{o}t}^{\text{2}}}{n_{\text{o}}}+\frac{\sigma_{os:t}^{2}}{n_{o}n_{s}}+\frac{\sigma_{tv}^{2}}{n_{v}}+\frac{\sigma_{ti:v}^{2}}{n_{i}n_{v}}+\frac{\sigma_{sv:t}^{2}}{n_{s}n_{v}}+\frac{\sigma_{si:tv}^{2}}{n_{s}n_{i}n_{v}}+\frac{\sigma_{otv}^{2}}{n_{o}n_{v}}+\frac{\sigma_{oti:v}^{2}}{n_{o}n_{i}n_{v}}+\frac{\sigma_{osv:t}^{2}}{n_{o}n_{s}n_{v}}+\frac{\sigma_{osi:tv,e}^{2}}{n_{o}n_{s}n_{i}n_{v}}-\lambda cn_{s}n_{i}n_{\text{v}}n_{o}+\lambda B \end{array}$$

$$\frac{\partial F}{\partial n_{s}}=-\frac{\sigma_{s:t}^{2}}{n_{s}^{2}}-\frac{\sigma_{os:t}^{2}}{n_{s}^{2}n_{o}}-\frac{\sigma_{sv:t}^{2}}{n_{s}^{2}n_{v}}-\frac{\sigma_{si:tv}^{2}}{n_{s}^{2}{n_{i}n}_{v}}-\frac{\sigma_{osv:t}^{2}}{n_{s}^{2}{n_{o}n}_{v}}-\frac{\sigma_{osi:tv,e}^{2}}{n_{s}^{2}{n_{i}n_{v}n}_{o}}-\lambda cn_{i}n_{v}n_{o}$$ (27)

$$\frac{\partial F}{\partial n_{i}}=-\frac{\sigma_{ti:v}^{2}}{n_{i}^{2}n_{v}}-\frac{\sigma_{si:tv}^{2}}{n_{i}^{2}n_{s}n_{v}}-\frac{\sigma_{oti:v}^{2}}{n_{i}^{2}n_{o}n_{v}}-\frac{\sigma_{osi:tv,e}^{2}}{n_{i}^{2}n_{s}{n_{o}n}_{v}}-\lambda cn_{s}n_{v}n_{o}$$ (28)

$$\frac{\partial F}{\partial \lambda }=-cn_{i}n_{s}n_{v}n_{o}+B$$ (29)

Let $\frac{\partial F}{\partial_{n_{s}}}$, $\frac{\partial F}{\partial_{n_{i}}}$, $\frac{\partial F}{\partial \lambda }$ are equal to 0:

Let (27) = 0, and get:

$$\lambda cn_{s}^{2}n_{i}^{2}n_{v}^{2}n_{o}^{2}=-n_{i}n_{v}n_{o}\sigma_{s:t}^{2}-n_{i}n_{v}\sigma_{os:t}^{2}-n_{i}n_{o}\sigma_{sv:t}^{2}-n_{o}\sigma_{si:tv}^{2}-n_{i}\sigma_{osv:t}^{2}-\sigma_{osi:tv,e}^{2}$$ (30)

Let (28) = 0, and get:

$$\lambda cn_{s}^{2}n_{i}^{2}n_{v}^{2}n_{o}^{2}=-n_{s}n_{o}\sigma_{ti:v}^{2}-n_{o}\sigma_{si:tv}^{2}-n_{s}\sigma_{oti:v}^{2}-\sigma_{osi:tv,e}^{2}$$ (31)

Let (29) = 0, and get:

$$B=cn_{i}n_{s}n_{v}n_{o},\frac{B}{c}=n_{i}n_{s}n_{v}n_{o}$$ (32)

Let (30) = (31), eliminate *λ*, and get:

$$n_{i}n_{v}n_{o}\sigma_{s:t}^{2}+n_{i}n_{v}\sigma_{os:t}^{2}+n_{i}n_{o}\sigma_{sv:t}^{2}+n_{o}\sigma_{si:tv}^{2}+n_{i}\sigma_{osv:t}^{2}+\sigma_{osi:tv,e}^{2}=n_{s}n_{o}\sigma_{ti:v}^{2}+n_{o}\sigma_{si:tv}^{2}+n_{s}\sigma_{oti:v}^{2}+\sigma_{osi:tv,e}^{2}$$

$$n_{i}n_{v}n_{o}\sigma_{s:t}^{2}+n_{i}n_{v}\sigma_{os:t}^{2}+n_{i}n_{o}\sigma_{sv:t}^{2}+n_{i}\sigma_{osv:t}^{2}=n_{s}n_{o}\sigma_{ti:v}^{2}+n_{s}\sigma_{oti:v}^{2}$$

$$n_{i}(n_{v}n_{o}\sigma_{s:t}^{2}+n_{v}\sigma_{os:t}^{2}+n_{o}\sigma_{sv:t}^{2}+\sigma_{osv:t}^{2})=n_{s}(n_{o}\sigma_{ti:v}^{2}+\sigma_{oti:v}^{2})$$ (33)

Jointly established by (32) and (33):

$$\left\{\begin{array}{l} \frac{B}{c}=n_{i}n_{s}n_{v}n_{o}\\ n_{i}(n_{v}n_{o}\sigma_{s:t}^{2}+n_{v}\sigma_{os:t}^{2}+n_{o}\sigma_{sv:t}^{2}+\sigma_{osv:t}^{2})=n_{s}(n_{o}\sigma_{ti:v}^{2}+\sigma_{oti:v}^{2}) \end{array}\right.$$

Results are showed:

$$n_{s}=\sqrt{\frac{\left(n_{v}n_{o}\sigma_{s:t}^{2}+n_{v}\sigma_{os:t}^{2}+n_{o}\sigma_{sv:t}^{2}+\sigma_{osv:t}^{2}\right)}{n_{v}n_{o}\text{(}n_{o}\sigma_{ti:v}^{2}+\sigma_{oti:v}^{2})}\frac{B}{c}}$$ (34)

$$n_{i}=\sqrt{\frac{n_{o}\sigma_{ti:v}^{2}+\sigma_{oti:v}^{2}}{n_{v}n_{o}\left(n_{v}n_{o}\sigma_{s:t}^{2}+n_{v}\sigma_{os:t}^{2}+n_{o}\sigma_{sv:t}^{2}+\sigma_{osv:t}^{2}\right)}\frac{B}{c}}$$ (35)

In Eq (34), *n*_*s*_ denotes the optimal number of students for *(s*:*t)* × *(i*:*v)* ×*o* design. In Eq (35), *n*_*i*_ represents the optimal number of items for *(s*:*t)* × *(i*:*v)* ×*o* design.

### 2.4 Operation steps of estimating the optimal sample size

The operation steps of estimating the optimal sample size for the Lagrange multiplier method in this article are as follows:

Step 1: In order to calculate the optimal sample size for each design, these optimal sample equations (*n*_*s*_, *n*_*i*_) need be derived according to different generalizability designs using the Lagrange multiplier method as above.
Step 2: After deriving the optimal sample Equations (*n*_*s*_, *n*_*i*_) for each design, the corresponding variance components needed be calculated by urGENOVA software [26].
Step 3: After obtaining the calculated variance component and providing other statistics such as *n*, *c* and *B*, according to step 1 and step 2 the optimal sample of *n*_*s*_ and *n*_*i*_ can be got.
Step 4: However, some results are non-integer values with decimals, which is unreasonable. Therefore, those results with decimals need to be rounded.

## 3 Empirical study

### 3.1 Data source

The analysis data used came from teaching ability evaluation of college teachers. The data was obtained through a questionnaire survey, which was conducted in two semesters. The first test was conducted at the end of the first semester (December in 2022) and the second test was conducted at the beginning of the second semester (March in 2023). The participants in this test were a total of 530 students from three colleges in Guangdong Province in China. The students who participated in the test evaluated their teachers (a total of 19 teachers). The Teachers’ Teaching Level Evaluation Scale for Colleges (TTLES-C) [27] was used to evaluate the teaching ability of college teachers. The TTLES-C consists of 25 items, divided into 5 dimensions, with each 5 items being a dimension. These dimensions include teaching methods, teaching content, teaching attitudes, teaching organizations, and teaching effects.

Before the formal investigation, with the written consent of the students, anonymity was also processed, which meet ethical requirements. Training was provided to the students on how to answer questions before formal surveys. Informed consent for behavioral experiment was given every student and teacher. The participants face to face were told that each of them needed to verify their approval of the questionnaire survey. Informed consent was obtained from all students and teachers. There were no obstacles to their participation because all the students and teachers are willing to participate. This study was approved by the South China Normal University (SCNU) research ethics board (Institutional Review Board) who approved the experiments, including any relevant details and confirmed that all experiments were performed in accordance with relevant guidelines and regulations. Research staffs were trained before they administered the survey. Data were collected during the class in 45 minutes using a survey administered to all students.

Some generalizability designs were considered such as the *(s*:*t)* ×*i* design, *(s*:*t)* × *(i*:*v)* design and *(s*:*t)* × *(i*:*v)* ×*o* design. Among them, *t* is the object of measurement, and *i*, *s*, *v*, and o are the facets of measurement. As mentioned above, in this article *t* represents the teachers, *i* represents the items, *s* represents the students, *v* represents the dimensions of the items, and *o* represents the occasions. The main purpose of this study is to present the application of Lagrange multiplier method to solve the optimal sample size of different generalizability designs under budget constraints. According to the operation steps of estimating the optimal sample size for the Lagrange multiplier method, the optimal sample size for the *(s*:*t) ×i*, *(s*:*t) × (i*:*v)*, and *(s*:*t) × (i*:*v) ×o* design can be estimated respectively.

### 3.2 Estimating the optimal sample size for the *(s*:*t) ×i* design

In the design of teaching ability evaluation of college teachers, the number of students being evaluated need be considered for generalizability design, but the students (*s*) are nested in the teachers (*t*) and crossed with the items (*i*), which is the *(s*:*t) × i* design. The estimated variance components for the *(s*:*t) ×i* design are shown in Table 1.

**Table 1:** The estimated variance components for the *(s*:*t) ×i* design.

	*t*	*s*:*t*	*i*	*ti*	*si*:*t*
*σ* ^2^	0.11059	0.26909	0.02702	0.02432	0.44349

According to Eqs (12) and (13), it can be calculated as *n*_*s*_ = 262.97048, *n*_*i*_ = 23.76693. In practical operation, the number of students *n*_*s*_ and the number of items *n*_*i*_ should be integers, and the calculated results of *n*_*s*_ and *n*_*i*_ should be rounded to the nearest 263 and 24. In the *(s*:*t) ×i* design, when *n*_*s*_ = 263 and *n*_*i*_ = 24, the relative error value of the design is *σ*^2^(*δ*) = 0.00151, and the optimal generalizability coefficient is *Eρ*^2^ = 0.98650. In the *(s*:*t) ×i* design, 263 students are selected, meaning that each teacher is evaluated by approximately 14 students. The item is set to 24 items. The optimal generalizability coefficient can be obtained, which means that the relative error variance of the measurement is the smallest and the reliability is the highest.

### 3.3 Estimating the optimal sample size for the *(s*:*t) × (i*:*v)* design

When designing tests, if the impact of item dimensions on test results is considered, dimensional analysis of tests can make the analysis results more specific and comprehensive [28]. In the process of teaching ability evaluation, the student (*s*) is nested within the teacher (*t*), and the item (*i*) is nested within the dimension (*v*), which is the *(s*:*t) × (i*:*v)* design. The estimated variance components for the *(s*:*t) × (i*:*v)* design are shown in Table 2.

**Table 2:** The estimated variance components for the *(s*:*t) × (i*:*v)* design.

	*t*	*s*:*t*	*v*	*i*:*v*	*tv*	*ti*:*v*	*sv*:*t*	*si*:*tv*
*σ* ^2^	0.10884	0.25595	0.02005	0.01032	0.01045	0.01561	0.07887	0.37777

According to Eqs (23) and (24), assuming *n*_*v*_ = 5, it can be calculated as *n*_*s*_ = 329.83942, *n*_*i*_ = 3.78972. In the process of teaching ability evaluation, the number of students *n*_*s*_ and the number of items *n*_*i*_ should be integers, so the calculated results of *n*_*s*_ and *n*_*i*_ should be rounded to 330 and 4. In the *(s*:*t)* × *(i*:*v)* design, when *n*_*s*_ = 330 and *n*_*i*_ = 4, the relative error variation of the design is *σ*^2^(*δ*) = 0.00325, and the optimal generalizability coefficient is *Eρ*^2^ = 0.97293. In the *(s*:*t)* × *(i*:*v)* design, when the dimension of the items is set to 5, the number of students is 330, which means that each teacher is evaluated by approximately 17 students. The item is set to 20 items, which means that each dimension of the evaluation items is set to approximately 4 items.

### 3.4 Estimating the optimal sample size for the *(s*:*t) × (i*:*v) ×o* design

In the classical test theory, retest reliability is one of the important indicators for the consistency of test results. As the name suggests, retesting refers to the application of the same test method to test the same group of subjects twice, and the reliability of retesting reflects the stability and consistency of the test over time [16, 25, 29]. When using generalizability theory for measurement research, the number of tests is also an important factor to consider. In the teaching ability evaluation of college teachers, in addition to considering the size of the test population, the number of evaluation items, and the dimensions of evaluation items, whether to use the same test items for a second test is also one of the issues that researchers need to weigh. To some extent, the presentation of retest results can make the test results more convincing. However, an increase in the number of tests means an increase in test costs [30]. Taking student evaluation as an example, in the evaluation process, evaluation at multiple time points or in multiple occasions can be included in the generalizability design, which means retesting. The evaluation student (*s*) is nested in the teacher (*t*), and the item *i* is nested in the dimension (*v*), the two are crossed with the occasion (*o*), which is the *(s*:*t) × (i*:*v) ×o*) design. The estimated variance components for the *(s*:*t) × (i*:*v)* design are shown in Table 3.

**Table 3:** The estimated variance components for the *(s*:*t) × (i*:*v) ×o* design.

*σ* ^2^	***o***	***t***	***s*:*t***	***v***	***i*:*v***	***ot***	***os*:*t***	***ov***	***oi*:*v***
	0.00147	0.08300	0.00288	0.02021	0.00850	0.02704	0.28058	-0.00018	0.00160
*σ* ^2^	***tv***	***ti*:*v***	***sv*:*t***	***si*:*tv***	***otv***	***oti*:*v***	***osv*:*t***	***osi*:*tv***	
	0.00692	0.01110	-0.00224	-0.00366	0.00059	0.00602	0.09078	0.40095	

According to Eqs (34) and (35), assuming *n*_*v*_ = 5, *n*_*o*_ = 2, it can be calculated as *n*_*s*_ = 183.35696, *n*_*i*_ = 3.40865. In practical operation, the number of students *n*_*s*_ and the number of items *n*_*i*_ should be integers, and the results of *n*_*s*_ and *n*_*i*_ must be rounded to 183 and 3. In the *(s*:*t) × (i*:*v) ×o* design, when *n*_*s*_ = 183 and *n*_*i*_ = 3, the relative error variance of the design is *σ*^2^(*δ*) = 0.01640, and the optimal generalizability coefficient is *Eρ*^2^ = 0.83501. In the *(s*:*t) × (i*:*v) ×o* design, when the dimension of the evaluation items is set to *n*_*v*_ = 5 and *n*_*o*_ = 2, the number of students is 183, which means that each teacher is evaluated by approximately 10 students. The item is set to 15 items, which means that each dimension of the evaluation items is set to approximately 3 items.

### 3.5 The optimal generalizability design

In this paper, The Lagrange multiplier method is applied to relatively more complex mixed generalizability design that includes both crossed design and nested design. The relatively more complex mixed generalizability design with four facets such as the *(s*:*t) × (i*:*v) ×o* design extend these researches of Marcoulides [14, 18] and Meyer et al. [7]. The research results of three generalizability designs that presented in this article are shown in Table 4.

**Table 4:** The research results of three generalizability designs.

Design	*σ*^2^(*δ*)	*Eρ* ^2^	*n*_*i*_	*n*_*s*_	* $n_{i}^{\prime }$ *	* $n_{s}^{\prime }$ *
***(s*:*t) ×i***	0.00151	0.98650	24	263	5	14
***(s*:*t) × (i*:*v)***	0.00325	0.97293	20	330	4	17
***(s*:*t) × (i*:*v) ×o***	0.01640	0.83501	15	183	3	10

From Table 4, the optimal generalizability coefficient for the *(s*:*t) × i* design is 0.98650; the optimal generalizability coefficient for the *(s*:*t) × (i*:*v)* design is 0.97293; the optimal generalizability coefficient for the *(s*:*t) × (i*:*v) ×o* design is 0.83501. Although the *(s*:*t) × (i*:*v) ×o* involves the other occasion (*o*) facet, but the optimal generalizability coefficient is the lowest among the three designs. On the premise of having a high generalizability coefficient, the *(s*:*t) × i* design and *(s*:*t) × (i*:*v)* design both exceed 0.90 which is often considered as the cut-off value for a reliability indicator. But the optimal generalizability coefficient of *(s*:*t) × (i*:*v) ×o* design is lower than 0.90. Therefore, it can be seen from Table 4 that the results of *(s*:*t) ×i* and *(s*:*t) × (i*:*v)* seem more reasonable.

In Table 4, $n_{i}^{\prime }$ represents the number of items included in each dimension and $n_{s}^{\prime }$ represents the average number of students evaluating each teacher. The $n_{i}^{\prime }$ and $n_{s}^{\prime }$ in the *(s*:*t) × i* design are 5 and 14; The $n_{i}^{\prime }$ and $n_{s}^{\prime }$ in the *(s*:*t) × (i*:*v)* design are 4 and 17; The $n_{i}^{\prime }$ and $n_{s}^{\prime }$ in the *(s*:*t) × (i*:*v) ×o* design are 3 and 10. If the actual situation is considered, the more items and students there are, the more reliable the estimated reliability may be. Therefore, undoubtedly the *(s*:*t) × i* design and *(s*:*t) × (i*:*v)* design are more reasonable.

Comparing with the *(s*:*t) × i* design, the *(s*:*t) × (i*:*v)* design examined more factors. For the *(s*:*t) × (i*:*v)* design, more consideration was given to the factor of dimension *v*. The *(s*:*t) × (i*:*v)* design not only considers evaluation students and evaluation items, but also considers the dimensions of the evaluation items, allowing for a more comprehensive analysis of the results.

In this paper, actual cost is c*n*_s_*n*_*i*_, *c* = 0.4. According to Table 4, the cost required for the *(s*:*t) × i* design is 2524.8 yuan (0.4×263×24), while the cost required for the *(s*:*t) × (i*:*v)* design is 2640 yuan (0.4×330×20), and the costs of the two designs are relatively similar and the budget cost is relatively reasonable. If the design has a high generalizability coefficient and can consider more facets, the *(s*:*t) × (i*:*v)* design seems to be more persuasive. From the perspective of generalizability design, this article believes that the *(s*:*t) × (i*:*v)* design belongs to the optimal generalizability design with budget constraints.

### 3.6 The originality and limitations of this study

The originality of this study is as follows: (1) This study firstly discussed that how is the unified formula of Lagrange multiplier method derived very meticulously and step-by-step, and how is the Lagrange multiplier method applied to estimate the optimal sample size under budget constraints for a relatively complex design with four facets in generalizability theory. (2) By conducting generalizability research and estimating the optimal sample size under budget constraints, the optimal generalizability design can be inferred effectively. Under budget constraints, taking into account the actual situation, the *(s*:*t) × (i*:*v)* design belongs to the optimal generalizability design.

For this paper, there are some following shortages: (1) Only three designs were selected to derive the formulas, and there are still many designs that can be discussed in practice. (2) As an example, only teaching ability evaluation of college teachers was selected. In fact, there are many examples that can also be used in the derived formulas. However, unfortunately those other examples were not discussed in this article. People can continue to study these issues in the future. (3) The Lagrange multiplier method has a long history and has many applications in obtaining numerical solutions for nonsmooth constrained optimization problems. This-type of method also has certain relationships with other first-order methods such as augmented Lagrangian method, proximal point method, alternating direction method of multipliers [31, 32]. However, the author only recalled the traditional constructing method, while the advanced constructing methods are neglected. (4) Although the title mentions the Lagrange multiplier method, the paper only discusses the traditional Lagrange function. It is recommended that future work explore methods like the augmented Lagrange multiplier method and the alternating direction method of multipliers for the problem addressed in this paper. In future, it’s better to make a comparison of the results with other methods and further analysis.

## 4 Conclusions and implications

By using the Lagrange multiplier method and the estimated variance components of different generalizability designs, the optimal sample size can be derived to minimize the relative error variation and maximize the generalizability coefficient under budget constraints. The Lagrange multiplier method can be effectively applied to the different generalizability designs under budget constraints, which indicates that the Lagrange multiplier method has new roles. According to empirical study, for some examples through three designs in teaching ability evaluation of college teachers, the equation for calculating the optimal sample size is derived using the Lagrange multiplier method.

By conducting generalizability research and estimating the optimal sample size under budget constraints, the optimal design generalizability under budget constraints can be inferred. As an example, in the teaching ability evaluation of college teachers in China, comparing with the *(s*:*t)* ×*i* design and *(s*:*t)* × *(i*:*v)* ×*o* design, the *(s*:*t) × (i*:*v)* design belongs to the optimal generalizability design under budget constraints if taking into account the actual situation. As for the *(s*:*t) × (i*:*v)* design, the optimal sample size of students is 17 for each teacher and the optimal sample size of items is 4 for each dimension by using the derived formula of the optimal sample size.

## Supporting information

S1 File This file includes (s: t)×i.doc, (s: t)×i_output.doc, (s: t)×(i: v).doc, (s: t)×(i: v) _output.doc, (s: t) ×(i: v)×o.doc, (s: t)×(i: v)×o_output.doc. For *(s*:*t)* ×*i* design, the (s: t)×i.doc is the program file in which contains data and the (s: t)×i_output.doc is result file. For *(s*:*t)* × *(i*:*v)* design, the (s: t)×(i: v).doc is the program file in which contains data and the (s: t) ×(i: v) _output.doc is result file. For *(s*:*t)* × *(i*:*v)* ×*o* design, the (s: t) ×(i: v)×o.doc is the program file in which contains data and the (s: t)×(i: v)×o_output.doc is result file.
(ZIP)
