# Supplementary material for: New roles of Lagrange multiplier method in generalizability theory: Inference of estimating the optimal sample size for teaching ability evaluation of college teachers
Source: PLoS One. 2024 Oct 17;19(10):e0307710. doi: 10.1371/journal.pone.0307710 (PMC11486427; doi:10.1371/journal.pone.0307710)
Supplement: S1 File — For (s:t) ×i design, the (s: t)×i.doc is the program file in which contains data and the (s: t)×i_output.doc is result file. For (s:t) × (i:v) design, the (s: t)×(i: v).doc is the program file in which contains data and the (s: t) ×(i: v) _output.doc is result file. For (s:t) × (i:v) ×o design, the (s: t) ×(i: v)×o.doc is the program file in which contains data and the (s: t)×(i: v)×o_output.doc is result file. (ZIP) [file pone.0307710.s001.zip › 2024-8-19 supporting information files/2024-8-19 supporting information files/(sú║t)x(iú║v).docx]

GSTUDY (s:t)x(i:v) Design

OPTIONS NREC 5 "*.out" EMS SECI .8 NOBANNER TIME

EFFECT t 19

EFFECT * s:t 22 25 25 31 60 19 25 29 35 17 22 64 27 26 20 21 21 22 19

EFFECT v 5

EFFECT i:v 5 5 5 5 5

FORMAT 0 1

PROCESS

4434334434443433444334334

5555544534555553435354334

4454333333444443334344334

3543543434355432435454344

4444434543445443444434544

5555552334525232134545321

5213433224234553535212113

4344334353443433434434333

5545454354455445455544555

5434434545554434444543444

5555354335555553555354444

5232234421555543334344334

5434544445343553445544554

4453444332555554333534433

4444433444444444445333214

5545555554555555455444444

4554554534435345453344534

5454555555445555555545554

5555111111555511115123111

4433322322433333344333333

4454334443433445444545454

4455445544554454545445544

5555555545555555555555555

4344444422555533432343333

5555555545555544545554444

5544454555555554444445545

5534343343554543344344333

3335455444555555444444444

5555445444554555444444445

4345545433444545433444444

5555555555555555554355544

5444444444544444555444444

5555555555555555544555555

4544554445454533445445445

5444455444543334445554433

5544443544544544555544455

5454444444555554334454455

5555444443455555554444444

4455555553455553544354444

4334434431444444434433333

5552355353355534545444444

3332325322555552324555555

5544345442544554544355444

5532213341555543433344224

3435344433555543555355333

3435344433555544555355333

5354555454543334551223455

1234444445555554554544444

5555545545545545445544444

4444433323445433333333333

3444333333333334333333333

4343434343434343434343434

3333333355543334224544433

4444443453453455354354354

3434444444544543453344553

1223412444522345434245215

5445444454555554554444444

4345534522544532334534434

4343334444344444444444444

5444444454444444445444544

5555545555455555554455555

5554545455545455555555555

3444444444444443334344343

3334445545454533544322232

3444444453454544434434443

5545345434534444452344554

5454345354354444555435543

5435434451324234322324535

5435455454355445454554555

5444434444444444444444444

3343334432343433334333333

4445554334553555334344335

4554544444434544345344445

4444445545545555444344445

3434343434343434343434343

5555554553555545344344533

4444444443455544344344444

5454445555445555545555545

4544543433455455334344333

4543445443344444444444444

3444444444444443334344333

5455545455545555555554445

5445444455555545544444554

4453454445445445444543444

5454545455555555455454555

4454544544454545454545455

5555455554555555555555455

4444555554445444454555444

5544544544454445454445555

3333332333333333332233333

4444444444344444444444444

3444444444444444444444444

3434443334443334434254224

3344333433334343434333434

4555544443444444344444444

5555555535555555555555555

5554454444555555555555555

3444444434444444545444445

4555554544555554444455545

5555455554555555544455555

4444555555544444444555555

3333324232332332222323332

4444434532444345324433434

4455445445454555444455455

4444444444444435354334445

4555555555555555555555555

4555555544445555553332255

4545555555555555555555555

5445455555555454545554444

3333334444433444455555555

4444444343444444443334434

4454444454545455544344434

5444444445455555535554433

3211231222133121225544554

4544444455444454444445455

3444344434343333233333343

4555555454445554444555554

3333333333333331333333333

3434343434343343323133333

5444444443455544344444443

5544455453454545554555445

2333544444444444444444435

4455544535555555444443333

5555555555555555555455555

4344443443444444444344444

5554433444555555433355555

5534554555344445154545335

5553344553455545335343445

4553543543454545423454345

4443323333323333233232333

3344433333343344444333443

5555555555544534454544535

4455455554545454455555554

5444333444445555443334455

5444444444443335554433333

4445444545444354333342434

5544455333445553455443333

3444444444433434334444444

4444444444444444444444444

3444444444444444433444444

3333333333444333333333333

5545454544555555554435555

5555454555445445555455545

5555555445445455554555455

4555555544555555555445555

4445445444555444445555544

5554555554444555555444444

4445545545555555455455333

3445545555554444444455555

4444344444445444444444323

3443555554454555444444455

4434444354444444444544444

4453435535555544224244333

4554454552444555334344455

4444444444444444444333333

5544555555545554554444444

5554554555444344434444444

3333333343455444444444333

4444344433344444344444444

5545455554445555545544344

5555544555544454545454544

4345544344344344443444344

4444444443435443445433444

5411531515355115513314315

2312321221222222122223112

5443444454444554343333333

4444444443433444343344334

5443434444444453433434333

5455553553335443333354333

4445553344444554343344334

5444343444444544333444333

5554443353455544553333334

4345334232434445454433443

4444455454444454445444445

3243133341342534122223332

3243133341342534122223332

1555525555255154555555555

5555555555555555455344444

5555455555455545554455445

4445445444445444444444444

5444343355443544454335434

5555555554544555555455544

2535545522455553545344435

5555555554555555545455445

4445545534555554545455445

5554544433444454344345344

5455544544545455454345344

4444444544444444454345344

4333434344343433242125434

3333333333443335543444335

5445555543445554444455434

3445555543454555444455444

5555455535555553554445445

5555555543555555544455555

3233322334332343332343432

3433434434443444343344434

4444444454455534444444444

4353444353454445433344553

5553545551445543543455434

4353444355443544354535455

3453344445555433443543454

5544455544445454453455545

3333333334434343443434343

4545455544454545434443545

5555555545555555455555555

4545445545334544545554333

5545355531555555353345335

3333333443343433333233323

5554454545454454334445544

4333332331233432223344343

4444444444444444444433444

5554554554554545544555555

4444444443455543334244445

4444544444444434455544444

4444434433444445555545555

3334444445555434234422223

3233332333232434333334443

4333334342233532323333333

4223223443233323224334433

3334342553333433445233333

4444434444344544334334434

5444445444444544444444444

3344333454445444333433343

4444444434444444444444444

4444443444444443333344444

5554545534444543445344445

4444444444344443344434444

4334433544445534444433333

3333333443334533344434343

3334345554444234544343443

4444333435444324444444444

4344432444133432222233333

5455555455554555555555554

5555554544545544555554545

4443345554444434445543455

3334434424333343332123334

4445334335544545343343434

4333334443444434433324324

4243324344432424344434334

4555554554555455555554445

5455555555455355555555535

5555555554555554444555555

4555454335555544454544555

4333232233333323333233222

3453434433444434354544444

5445444413555534434434444

5555554443555555554444433

3445344352445545434434433

3444333343434434433334334

3445433432345545444554445

4444543443444445444454444

5555444453555545444444335

4555555545555544454434435

3444344342445555554333333

4555453225555554354545244

5445444555545555444544444

4444344444444434444344444

2112211232121132213222121

4345435554444544444444445

3445453345445545554344333

5545444454545545545445555

4555455434555555454534435

3454443354545444534345454

5444443454555545445445534

4555344455555545455445555

4334444443555454434324333

5434454443455554335444334

4444444444444444445544434

4545544445545445454444544

4555454545444555545454455

5555555445544555455554555

5555454434445554455455555

4544533434444455543344445

3444442444444545443223322

4545455544334443533543344

4445534323444545545444445

4554444554555554443454444

4444335534444343344434544

5555454455545455554344454

5545335355554453455344555

4345544443444454444445445

4555554455555555555555555

3455544455454544544544545

3555555544353355555543445

5554445544555554444344344

5554445544555554444344344

4444554445455554434333434

4444444455433444342233444

4534554555454555454545554

3443545455534555545454535

4553534555345455554355545

3454445435444534555545455

5435535444554355555455555

4434545453454455554355545

4545554554545454544355555

5553454545454545344544545

4555555555455555554555545

5455554535445455555555555

5455433455544344555545455

4444444444444444443434445

4444344543344454454435445

5554545555455555555555555

2345432123453212321234543

4444433443344333334333222

5454445543445544554555444

2123334341322234343433333

5555555555555555444555444

3454553445435453454455354

4555435454545544344343555

5554545454545434355544544

5222334422343443223234332

5443454445455544243333434

4444433443334433344234434

4444334343443443434344324

5554545545555554455454455

4455443553444544453344334

3334324422354534324144344

4444443344444444342344444

4445455454444444543445445

3444444444544533435234334

5455444444555324443422444

4344445344322523532132424

2324444433244442234244444

5344443343344243444443333

4554342143444542342244212

3222222333133533243223223

5555445434444444444344434

4555555533555553545355355

4444434444333543425244444

4235545442545542225344244

4434345445444444425244333

4544545344454444345244445

4344434444433444444444434

4344334332454434423233544

5444344435434444444444555

4444443333444444444444444

5355555555533555355535434

5555555354444543344344333

5344435355445543435433445

4544444344444443344344434

4445444544455254444453445

4543443344434544443334444

4444433334444333334434334

3333343323444433333323323

4434424233445533335424224

3444445424455334425444334

5444444234355454325134324

5554444344444443344344444

3344434443444434444333434

4454534554555454122133322

3343333333234433232234333

4444343344455544444333333

4444342434444442233343333

2453245121445522324122222

4333424453353524334143334

4444335524333523444245424

4254435445555534445234544

4444545443443434434243334

4433434244443444444333333

3334333242233434534333333

4334334455554545334233323

4344335432444443544544344

5444444334444543244244345

4444444442444444344433434

4444434333555444434344444

4445544442444554444454444

4333224252444432343211111

4443344345555555544344544

4444444444444444444244234

3341134331243412112111111

5434334344333434442333434

3344443321223323343423322

5234442224454444324224334

3322324211212432222221111

3545553522455443334333334

5555555545555545555455435

5444545544545455444444433

5555454555555555555555555

5554443435555354433453335

5555554555555555555554545

4444544443444444334333444

5555535545555554555444555

5554555555555555555445355

5555443342555544433333233

4444444444344444444333334

5453355452555553434333334

5555555555355555455444444

5444444454545554445433333

5555555543555554334333333

4443444444444434444433333

5443544445555553233244222

5444444545444544344433334

5555555533555555454555445

5555454522555554445335324

5445455544545545544455555

4444343434334444444433333

5554555554555554545534344

5555554455455554544433444

5555555555555554535334334

5435455555555443445334344

5554545554555544555455445

5555555553555554355555444

4444444444444444444433333

3555555553455553545455445

3443332253453232124223224

5555555555555555554555555

3443454342444443234234334

1344443321334544535433225

2444543433444543344344433

2444444443443444444444544

4444544442444434444444554

5555555555555555555555554

5454445443455443444454445

4444444444444444444354354

5555555555555555554445555

5554544455555553345544444

5444454544454455545445444

5545545455545554545454545

5545555555455554555554545

5455455454544445554455444

5434454544443444444533333

3445545552455432553344525

3554555542444552455344545

3444434453445543444444444

5334534333555233455344444

4554555555555544545444444

3444444432555554345455555

5555555555555555554445223

4555353255544555343224323

5555343353455555443324334

4554443333554544434433333

4444323344444544442245334

3555553354555555533344333

5554343343334444443333344

4555453352555555434455534

5555553343545544434344334

5555444334444444333333333

4555555555555555555455555

4555454453555555554433333

5555553353555555555355335

5555555555555555552333344

4555453322544555532334333

2443322311443443233233324

5455554454445545443334334

5555555555555555555353221

5555352252444553553144242

2222233322233223555445555

4445455555554545545455555

5444555444554444554455544

5555444453554555455444554

5445434355443454345454433

5444544554554334555555435

5554545454445455555545545

5554454555555445444345434

5554454555555554554445545

5555554455555555455455555

5555555554555555554455444

5454344444555554434343435

4544454445454454454554445

5555554555555554555544334

5555555555555554455444444

5545445434444544343444434

5555554555545554554454555

5443342353555554532455345

5444444454444544555544455

4554454544555544454544545

5555454545545553555544334

4545454454555445554555445

3555455534544544343344333

4443344343334443433443433

4555554443444454334455444

3333322333233333443332334

4445555544444455544455444

3222244423322522333223423

1111113222111111211122222

4444455532334433334443334

2111231212112111123311213

3321322232211222233322232

4444444332343433233234444

2311213313111123121121111

1221322312312112332111233

2444444334444343224234444

1222223233322333332333232

1222123132321233323344444

3211213213332211111111111

3444433443444444333434333

3444433443444444333434333

1111223313222122112232222

1111112112111112212211111

5445444454544443555434545

5555444554334455444455544

5554443214444544333344334

4545555545555545555555455

4544345455544534545445455

4555545545545544554545554

5555454455555445455444344

5545555555455454555555455

4334333332344543334335545

5454344444555554434343435

5445445554555455544445444

4444344444434444445555455

5555555455555554454555555

5443455554545554555543345

5555444555555555444445445

5445443433434544434433343

5555455455455554554555445

5355535555445535335555235

5444554445444544445444544

5545443434545554444454445

5444445454445555544444334

4555544544455445555454455

5555444444444555555354444

3333333444444343433323234

4554454444555554434344333

4453344322333444442244434

5555445544444454433445334

5555554454555555553455544

3333344444555554444433333

5555555544555555555555445

3555555555355555445555544

5555555555555555454355445

5555555544555553544444333

4444444433444544443434343

4444444433455554534444555

4555454554555555444534434

5555444452444444221344333

5554444454444444444444454

5555555555555552455535555

4333344344444242443343324

5555555555444444444555444

FINISH
