# Supplementary material for: New roles of Lagrange multiplier method in generalizability theory: Inference of estimating the optimal sample size for teaching ability evaluation of college teachers
Source: PLoS One. 2024 Oct 17;19(10):e0307710. doi: 10.1371/journal.pone.0307710 (PMC11486427; doi:10.1371/journal.pone.0307710)
Supplement: S1 File — For (s:t) ×i design, the (s: t)×i.doc is the program file in which contains data and the (s: t)×i_output.doc is result file. For (s:t) × (i:v) design, the (s: t)×(i: v).doc is the program file in which contains data and the (s: t) ×(i: v) _output.doc is result file. For (s:t) × (i:v) ×o design, the (s: t) ×(i: v)×o.doc is the program file in which contains data and the (s: t)×(i: v)×o_output.doc is result file. (ZIP) [file pone.0307710.s001.zip › 2024-8-19 supporting information files/2024-8-19 supporting information files/(sú║t)x(iú║v)_output.docx]

CONTROL CARDS FOR RUN 1

Control Cards File Name: 3.crd

(s:t)x(i:v) Design

GSTUDY (s:t)x(i:v) Design

OPTIONS NREC 5 "*.out" EMS SECI .8 NOBANNER TIME

EFFECT t 19

EFFECT * s:t 22 25 25 31 60 19 25 29 35 17 22 64 27 26 20 21 21 22 19

EFFECT v 5

EFFECT i:v 5 5 5 5 5

FORMAT 0 1

PROCESS

INPUT RECORDS FOR RUN 1

(s:t)x(i:v) Design

RECORD NUMBER 1:

4.000 4.000 3.000 4.000 3.000 3.000 4.000 4.000 3.000 4.000

4.000 4.000 3.000 4.000 3.000 3.000 4.000 4.000 4.000 3.000

3.000 4.000 3.000 3.000 4.000

RECORD NUMBER 2:

5.000 5.000 5.000 5.000 5.000 4.000 4.000 5.000 3.000 4.000

5.000 5.000 5.000 5.000 5.000 3.000 4.000 3.000 5.000 3.000

5.000 4.000 3.000 3.000 4.000

RECORD NUMBER 3:

4.000 4.000 5.000 4.000 3.000 3.000 3.000 3.000 3.000 3.000

4.000 4.000 4.000 4.000 4.000 3.000 3.000 3.000 4.000 3.000

4.000 4.000 3.000 3.000 4.000

RECORD NUMBER 4:

3.000 5.000 4.000 3.000 5.000 4.000 3.000 4.000 3.000 4.000

3.000 5.000 5.000 4.000 3.000 2.000 4.000 3.000 5.000 4.000

5.000 4.000 3.000 4.000 4.000

RECORD NUMBER 5:

4.000 4.000 4.000 4.000 4.000 3.000 4.000 5.000 4.000 3.000

4.000 4.000 5.000 4.000 4.000 3.000 4.000 4.000 4.000 4.000

3.000 4.000 5.000 4.000 4.000

MEANS FOR MAIN EFFECTS FOR RUN 1

(s:t)x(i:v) Design

Means for t

3.860 4.200 3.909 4.139 4.109 3.773 4.120 3.750 4.063 4.207

4.247 3.624 4.267 4.186 4.014 4.472 2.760 4.353 4.187

Means for s:t

3.560 4.280 3.560 3.840 3.960 3.560 2.960 3.520 4.520 4.080

4.400 3.440 4.200 3.840 3.640 4.640 4.120 4.760 2.560 3.040

4.000 4.440 4.960 3.560 4.680 4.560 3.760 4.240 4.440 4.000

4.800 4.200 4.920 4.280 4.080 4.400 4.320 4.400 4.400 3.480

4.160 3.760 4.200 3.400 4.000 4.040 3.960 4.120 4.560 3.360

3.160 3.520 3.480 4.040 3.960 3.120 4.400 3.720 3.800 4.160

4.840 4.800 3.720 3.640 3.920 4.080 4.200 3.400 4.480 4.000

3.200 4.040 4.120 4.320 3.480 4.360 4.000 4.680 3.880 3.960

3.680 4.720 4.480 4.160 4.720 4.440 4.880 4.400 4.440 2.880

3.960 3.960 3.440 3.360 4.080 4.920 4.760 4.040 4.600 4.800

4.520 2.640 3.640 4.480 3.960 4.960 4.320 4.920 4.600 4.000

3.760 4.200 4.320 2.640 4.280 3.320 4.600 2.920 3.160 4.040

4.520 3.840 4.240 4.960 3.840 4.360 4.200 4.160 4.080 2.920

3.440 4.560 4.640 4.040 3.840 3.840 4.040 3.760 4.000 3.880

3.120 4.640 4.680 4.720 4.800 4.400 4.560 4.480 4.480 3.840

4.360 4.000 3.840 4.200 3.760 4.560 4.240 3.600 3.800 4.520

4.560 3.840 3.880 3.080 1.880 3.800 3.680 3.720 3.880 3.920

3.760 4.000 3.640 4.240 2.720 2.720 4.400 4.680 4.720 4.120

3.960 4.760 4.080 4.800 4.520 4.040 4.360 4.040 3.280 3.480

4.360 4.360 4.600 4.760 2.920 3.600 4.120 3.920 4.200 4.120

4.000 4.440 3.360 4.320 4.920 4.200 4.200 3.080 4.320 2.920

3.920 4.720 3.920 4.120 4.240 3.520 3.040 3.040 2.920 3.360

3.760 4.120 3.600 3.960 3.760 4.240 3.840 3.720 3.400 3.760

3.760 3.000 4.840 4.680 4.120 3.080 3.840 3.400 3.360 4.720

4.760 4.800 4.480 2.720 3.840 3.960 4.440 3.840 3.440 4.000

4.040 4.320 4.480 3.800 4.200 4.440 3.880 1.720 4.160 4.040

4.560 4.440 4.080 4.320 4.600 3.760 4.040 4.040 4.360 4.560

4.760 4.560 4.040 3.520 4.000 4.080 4.360 3.840 4.520 4.360

4.120 4.880 4.400 4.400 4.320 4.320 4.080 3.720 4.560 4.360

4.440 4.320 4.520 4.360 4.560 4.400 4.840 4.720 4.400 3.960

4.040 4.880 2.920 3.280 4.400 2.840 4.760 4.200 4.280 4.400

2.960 3.920 3.520 3.560 4.680 4.000 3.320 3.760 4.240 3.760

3.920 3.280 3.360 3.560 3.280 2.600 4.080 4.520 3.720 3.720

3.760 4.080 3.800 3.480 4.080 3.840 4.440 4.080 4.040 3.840

4.160 3.800 3.520 3.080 3.440 3.800 3.640 3.960 3.640 3.600

3.000 3.760 3.400 2.840 3.400 3.600 4.080 3.720 3.520 3.160

3.680 3.800 3.800 3.760 3.880 4.120 2.720 4.200 3.800 2.080

3.480 2.880 3.360 2.040 3.720 4.760 4.240 4.920 4.080 4.880

3.800 4.720 4.800 3.800 3.760 3.960 4.640 4.080 4.160 3.720

3.760 3.960 4.680 4.160 4.600 3.600 4.560 4.440 4.480 4.240

4.680 4.680 3.800 4.560 2.920 4.960 3.480 3.360 3.680 3.880

4.000 4.960 4.200 4.000 4.880 4.440 4.360 4.640 4.800 4.480

3.880 4.040 4.240 3.960 3.840 4.560 4.240 4.560 3.840 4.000

3.800 3.640 4.160 3.680 4.320 4.080 3.720 4.920 4.280 4.520

4.480 3.800 2.880 4.160 4.440 3.760 3.240 4.680 4.440 4.520

4.040 4.400 4.640 4.400 4.680 4.840 4.760 4.120 4.360 4.640

4.680 4.040 4.760 4.040 4.360 4.480 4.480 4.560 4.000 3.520

4.200 2.960 4.400 2.760 1.440 3.640 1.600 2.200 3.400 1.600

1.920 3.480 2.480 2.560 1.600 3.560 3.560 1.840 1.240 4.280

4.360 3.680 4.800 4.360 4.640 4.480 4.800 3.560 4.120 4.480

4.160 4.840 4.440 4.600 3.760 4.720 4.320 4.280 4.280 4.240

4.560 4.400 3.280 4.120 3.440 4.160 4.680 3.800 4.840 4.680

4.760 4.400 3.800 4.200 4.440 3.680 4.200 4.760 3.400 4.520

Means for v

4.108 3.950 4.195 3.870 3.844

Means for i:v

4.126 4.121 4.138 4.130 4.023 3.998 4.023 4.011 3.968 3.751

4.132 4.206 4.234 4.306 4.098 3.964 3.896 3.892 3.960 3.638

3.853 3.974 3.755 3.655 3.985

ANOVA TABLE FOR RUN 1

(s:t)x(i:v) Design

-----------------------------------------------------------------------------

Effect df T SS MS VC

-----------------------------------------------------------------------------

t 18 212818.41265 1513.84140 84.10230 0.10884

s:t 511 216482.68000 3664.26735 7.17078 0.25595

v 4 211551.11057 246.53932 61.63483 0.02005

i:v 20 211678.51132 127.40075 6.37004 0.01032

tv 72 213255.33454 190.38257 2.64420 0.01045

ti:v 360 213673.73085 290.99556 0.80832 0.01561

sv:t 2044 218497.80000 1578.19811 0.77211 0.07887

si:tv 10220 222777.00000 3860.80369 0.37777 0.37777

-----------------------------------------------------------------------------

Mean 211304.57125

-----------------------------------------------------------------------------

Total 13249 11472.42875

-----------------------------------------------------------------------------

Grand Mean: 3.99343

STANDARD ERRORS AND CONFIDENCE INTERVALS FOR VARIANCE COMPONENTS FOR RUN 1

TING ET AL. INTERVALS

(s:t)x(i:v) Design

***Output not provided because design is unbalanced***

EXPECTED MEAN SQUARE EQUATIONS FOR RUN 1

(s:t)x(i:v) Design

EMS(t) = 1.000*VC(si:tv) + 5.000*VC(sv:t) + 27.584*VC(ti:v)

+ 137.920*VC(tv) + 25.000*VC(s:t)

+ 689.602*VC(t)

EMS(s:t) = 1.000*VC(si:tv) + 5.000*VC(sv:t) + 25.000*VC(s:t)

EMS(v) = 1.000*VC(si:tv) + 5.000*VC(sv:t) + 33.487*VC(ti:v)

+ 167.434*VC(tv) + 530.000*VC(i:v)

+2650.000*VC(v)

EMS(i:v) = 1.000*VC(si:tv) + 33.487*VC(ti:v) + 530.000*VC(i:v)

EMS(tv) = 1.000*VC(si:tv) + 5.000*VC(sv:t) + 27.584*VC(ti:v)

+ 137.920*VC(tv)

EMS(ti:v) = 1.000*VC(si:tv) + 27.584*VC(ti:v)

EMS(sv:t) = 1.000*VC(si:tv) + 5.000*VC(sv:t)

EMS(si:tv) = 1.000*VC(si:tv)

*** EMS matrix is upper diagonal***

Date and time at beginning of Run 1: Sun Jan 8 16:35:06 2017

Processor time for run: 0 seconds
