# Supplementary material for: New roles of Lagrange multiplier method in generalizability theory: Inference of estimating the optimal sample size for teaching ability evaluation of college teachers
Source: PLoS One. 2024 Oct 17;19(10):e0307710. doi: 10.1371/journal.pone.0307710 (PMC11486427; doi:10.1371/journal.pone.0307710)
Supplement: S1 File — For (s:t) ×i design, the (s: t)×i.doc is the program file in which contains data and the (s: t)×i_output.doc is result file. For (s:t) × (i:v) design, the (s: t)×(i: v).doc is the program file in which contains data and the (s: t) ×(i: v) _output.doc is result file. For (s:t) × (i:v) ×o design, the (s: t) ×(i: v)×o.doc is the program file in which contains data and the (s: t)×(i: v)×o_output.doc is result file. (ZIP) [file pone.0307710.s001.zip › 2024-8-19 supporting information files/2024-8-19 supporting information files/(sú║t)x(iú║v)xo.docx]

GSTUDY (s:t)x(i:v)xo Design

OPTIONS NREC 5 "*.out" EMS SECI .8 NOBANNER TIME

EFFECT o 2

EFFECT t 19

EFFECT * s:t 22 25 25 31 60 19 25 29 35 17 22 64 27 26 20 21 21 22 19

EFFECT v 5

EFFECT i:v 5 5 5 5 5

FORMAT 0 1

PROCESS

4555545444455555554454445

5554545355555545555455554

4444444443343433343433444

4554555545555455554545555

4545554555554454455545555

5444544444444544555544454

4334443443445443334444334

3454342235545545555555555

4455555545555332355533333

5555553553545555554555445

4443432343324445442233333

5555555554555555455554555

4254212452434311555415514

4555555554544555454554554

5554455455554435555554555

5544454454555545353355545

5545453354554545455344444

4555555454555555545455454

5555555453555554455355445

4444343344334443343435434

4445343344434553533333434

4444454454454544344355434

4444344334434343443444333

4444334432444342535543433

3444444443443443554443444

4555455533554553554355435

4555555445555554554555545

5555555555555454455555545

4444555444455554554555555

5455555444554555554544544

5555555544555554545554555

5553555515355553555553223

5344355444443555435444335

4455555522555553545553444

4554445444555553455455455

4344434444444444444444444

5455544433555553544354444

5545444433545544434455434

5455555433555453545555444

5443545445333551345333333

5445443533555543355444445

5455555523555444555455555

4444333334444443333333333

4444434333555543444353333

3544434334455553454544434

3314534344353433333233333

5555555445555554555555555

5535445532243553553533333

5345444453455545555444444

5445443535555544354433433

4445445554554555555455455

5555555534555555555555445

5555555553555555555544555

5545455443444554455444444

5354445533344544345444444

4434344313555544555443443

5345444424445543444443434

4332333323333443333333333

3333434422333432433423323

3444433433343444344434444

5444544444455544555455454

5445555555555555555455555

5445445534455553445543333

5555455555555555554555555

5445434423555543554445445

5555555555333334554555543

4444344443444443334433334

4344445431353433333233333

5555555544545545555543333

3333334432433434324333334

4555445534252322333233333

4445544535445554554555555

5443443433445534433334553

5455445455544544444544555

4545554545444545455444454

5555555555555554444454444

4554544545455454434443434

4555444545554445455555444

5555554545555555454455555

4444443434344444433344444

4444343443444444223234333

4544454545553552333344334

4455545455455454554545555

5544544532444444425555555

4454354533444554334344343

5544455544554555445445544

4444444454555555455555555

4443532444335545443333334

4545545545455545545544454

5555555555455555555555555

5545555545555555555555555

5555555543555555555455555

5555455544555554555555555

4445454554554554455544445

5545555534555555545555544

5555555555555555545555555

4455455444454554511144334

4443444434344454334443455

5555455554555555555555555

5555555545454555555555455

4343444343323322444444423

5554555544555545444554555

4545544445545445455445545

5455555455455543554354544

5555554455445444455445545

4433333334444443433344444

5355455544555555333555545

4455355533555555333345554

3345545443544555334454455

5545545554555454445455545

3343544555454554343444534

4545445544554554345455555

4454545444444445555544545

4455455555555554554454444

4545555454555555554454554

2222322222111113232211111

3355444543555554233344444

5332534535444432522343555

3244544552343444234444445

4322344444444442224244434

2322432452343323334244443

3322524555555433535343454

3333444435555553555544444

3443453534443552324234442

5434445444543435454454434

3343434444344554233244443

4434334444444454444454544

4333434545345552334243553

1245334435334442333344434

3434444444433443344433333

3343433434443443434333333

4442222224222223445243444

3355455555555555555555554

5555555553233212131211112

3323422433344332343234332

3433444443434343344343343

4533444434544443333344444

5443453434534444544454545

3334434545555542332343443

4444445554555443333443345

4344434444444443334344444

3432433444444433224233333

4344444553444543333254434

3333343434444333343443334

5445455555455554445333333

4344443443444443232244334

4455455444454544444444445

4545455435445554444434444

4334433334333433334333333

5555544545455454455554445

4545544454434544545445444

5555555545555554455555555

1212122332222131112221111

4434444444455544455455545

5345544434554454443355555

4444455555445453445555555

2412454214215323543232234

4444355554345444444553444

4544555545555445345444444

4444444543444544544434544

4433455554444443444444444

4543444454443444444444343

3543343525433544341144433

3223222231223211122233112

4555355453435545345433554

3121133422111111333222222

2212221122212222322122222

4343343441222322111111222

4443443233344334444535434

2132132331122122334422222

3424344332233422343323223

4533333343444343333344443

4534354322444452322233334

2444433443444342242334333

3334334343444441242134334

1121122215455443441554344

4545455553445553344255555

3345544434455543443343334

2112212112221111111111122

5555554433555441333154444

3235111312113131122212222

3343444344434344233443444

3332433434222333443344443

4344434434554544454544333

3434333434444333434343333

5544555555555455555555555

4444444454554544444444334

4444434434444444444434444

4334434434444543434334334

3223312343342235523433342

3444335433324341333434433

2455555443555544324433334

3355533334443333445444444

5333555535555353333454435

5445555453332224222222221

4444444334455553444453334

5455545555544555555454455

5545555545545455545545545

5555555555555555535555445

2444433425555434544443344

5444443443454444534344345

5545454554553555355555335

5455555553555554445354434

4344244232344442423233324

5455555445545555344444544

4555555555555554455555555

4454453435554554553555434

4434343343343444333333333

3332323322132423333423333

5444444433445443344334444

4544344433444443345344444

4234344433344433534334444

4333433434334433333344444

3444443444444433435344444

4113313332222323223344443

3233332454333531325223342

4433344443444444344444444

4444434344344343444343444

5555555555555555445555555

4555455545444555455555554

3434444544333544233434434

4111322343133433233433224

4444343434434433344344343

4334343353333443444334444

5555553553355553355344453

4445445555444544545443444

2313344324333343223323344

3323343432255433333332223

4344444453444544434344444

2322111113222131111211112

4445454544454555544554544

4544545544445545544454454

3321323332333343332332123

3323323333323433343323243

4444444432354443344433333

2332322234333222223212222

5445353443545555544444445

1343114511354421423444423

5555455453555555454555544

5455534334555555545554555

4555444443455555545555444

4555253114455545544535434

5555555454555555334255544

2334334333454433433433333

4444444443444443433333333

3455354343554555554544333

4455543444555552533334435

3545355344334534533455335

5455545544555555545555555

4445435555555334534355555

5555455544555545554555545

4455444544555545545444444

5454324253325535443355222

5455455553555555545555555

3553455554555554555544435

5555455543555555333334333

4555555354555555554545535

4434343434555553533222332

5545455453454555544555445

3545444324545553454343444

4354433433545554434454545

5455554544555555555445555

4555555452554535443435344

4455443535545555454555555

5444333332444533444443334

4444444443444443433443433

3553455555555553533453344

4444334433434434444434434

4444433334333232343343333

3345445454445535444445444

2224223323445454553555555

4554455535444454453455534

4555443453444445433444335

5444344444345433433233333

3433433333443334333322222

4554544455554553444455555

3553433425544445322234433

3345444443434244443444444

3433443244344443322433333

4444243443344444433433333

3444444445554444444344434

5555555555555555555555444

4545544523555544234244434

4545443454233553223444444

4544454345345444344444333

4454544333444442233333344

3245444555334344534344444

4554455535444454453455534

4444434334445344345445454

5555555454555454544555445

5555555555455554555554555

3555555555555555555555555

4444444343444442324333223

5554455454554444445544454

5455455433554554555554444

4555544115555555535455323

5545535545555554545535245

4445455554454534554545444

5555555515455553535555555

5545544555455555555555555

3131144333332533433233111

5455443345454454454544554

5554455545555555434454334

3344443532334543334443333

5455555555554555545545554

4545545545455454545445555

4545444455555554555455554

5554555445455445554455555

5444544454555554555554443

5443453334545543534544344

4444435554244335445353244

4435435431333255435213115

4445545543444444333344224

4444444433444545434343334

5455555554555545554554555

2234223244443141212121222

4442355224325415443333334

4442144341454444211224212

4444455433444444455344444

4444344344344343334344444

4344444432444443242444224

5554545443544343544444444

3113324233333423133331343

4445435444333343444244343

3422323343433344333333333

4422213323434323113333333

4432321323344323113343333

2224344344423243223434443

2243232232322324322223323

4134424422444344443444445

3344433422334444443344434

3444454443444554534345555

4355455553553444544444333

4333422333244333433234323

4332332222242422332233322

4444434433444433333244444

4344444454444454454444444

2234434444444443443443434

4533435432555554335244434

5454453344525535342213123

3532245443434544243443434

2323224232234433343334323

5555454544535554443544454

4544444225234441244255544

1121122113112213122111111

5344344444334335344333333

4454324532455542444253222

4443444452454454234244535

5555555344555555435344444

4444544333334343544445554

4544454444444445334334444

2333332433222214332333223

4444444434444554545444444

4343444422444544333344224

4323334442333434422233333

4443324442555554433233324

3344444443334333344434434

4444445442434444444333434

4434444432444444344344424

2434444333333434434333444

4434434342344434224333333

4444444333333333333344444

5555555544555554555555355

5343445455533545525343545

4443434334344444333333333

4555555545555554444444444

4434444533444343443444544

4544544444444444444444444

4445444444444444444444444

4444444344444444333333333

4424444443444442224344444

4344444242424443224234445

5555555553552555555555345

4344444334445444524334444

5455555545554554435455334

5555455543555555545344334

4444545444534553334444434

5555544454453534444554455

5555555555555554555455444

4444443343444444443444444

4333432323554543434443333

5432555425555555425143322

5555544455555554554555444

4544454455454543444344434

3334443443343334233334333

4444444444444444344334334

4444444443344444444334334

4444544444444444344444444

4554544435455443333244323

5444455542555555435233334

3344434444444444444333334

5455455554555555554544344

5543455343444552332334223

5544444433454554334344444

5555554545555455445445444

4545555543555554444344434

4434433343445543545443334

5545535455545545555555555

4535533443555255324443434

4544543543454454335444445

5454544533555553345344334

3544444443445342344444344

4544444444454445444344544

3454434533454542344334334

3433333434444343433333334

3344432353542422434455413

2454334433455552554545555

4454555333555452435434445

3543443432345341322243334

4233344333322244343334443

4444554543454542255233444

4445544444333432445232334

4444444433444443444344444

2353545415555553535333555

5554555545355553555455335

2333433241244431444222314

5553355555555555555555555

5555555555555455555555555

5555555555555554555555555

4445444545555544544445555

5454455433555543454334433

5455544444445443344445444

3443444333343432343234433

4353233325233535334433322

3444524433343122544222434

3333423333323132422334323

3334454442544431434223333

3553551453533553142413323

4434443333333552434435222

5324343333334444422233244

5444344323444444444344444

5545554443344455454445435

4445444454444554453444444

4332323344434434334533333

4555554544555555543245344

4444343434333443333233333

3333334545333124334333444

3555353343445553443345333

5555453353545554534344334

4434343331443443333133123

5544544444444344333333444

5555452231545552322344122

3443433333335333334443444

1343245455415155122315555

5444443343445544434444444

5555555553555553334334334

4555554353553553444344444

4455555555455455455555555

3233444425343343444444444

3334344443434533434334343

5445555455445555554545545

5445445444544543534343443

3555554454555555334555555

5554545525445545355555334

5554555445545555555555555

2334443433333443343433333

4444444453545555555544344

5555554555553555435555555

5554355335544554345544333

5555555555554554545555445

4455455552455555354355535

5555555455435555555554535

4445445444555445445444454

4444444443444444444444444

2423344341444531311323222

3433334433333433333333333

4534555455554555555555445

5555544543555445544553545

2212221531222112112221333

1444443344344443444355344

3311413331223221123322222

3433321233344233533333433

4234333432333423222232423

3555554433445453333354333

3444443322444443223344333

4554545443543344544422423

3235343444422344543434443

4344454543443543434322222

5555543545244253442343445

3444343333435544333434334

3345354454554555555554545

2343555213335544443233323

3322333344233344223323232

3322333334443454344333332

3235452344112223412542231

2143142324213424334234223

2111121224211121332111211

4453455554545544554545544

3334344343344334343334334

5245544544545555545555545

4344344424432244444444444

4332344323342123333432233

4555455444555554554455444

4444353343444431524322323

4555455433455544344555555

3545454533555555544555335

5555455454544444454455544

3323442334333243533234333

4444343443444444443444444

5554343452555535525554545

4445455554555555445443333

5555554555555554555555445

4545455542343553324355335

5555555553555554553555545

5454455554544444554445443

3344343443444443344444444

4512444442333433322323223

3334343444344333333333333

4333422144211113112323543

3445444545445554554445555

5443355544554554434555454

4543344444444434434443334

5555555544455555455555555

5545454555445455434443334

5554545554554555445555445

4554445523345455345534334

5553455553555555521155334

2343444344434344555544444

4453523544434355455345543

5544455444444441124244444

5555555553555555555555555

5454354543444454323233444

4344454453555544443355544

5454455551555551524545411

5545555555555555444455555

5544544444454444445444444

5555555553555554435554334

4444344424324354353344333

5555554445445554434444434

5545454554545545555555455

4434334434443433444334334

5555544534555553435354334

4454333333444443334344334

3543543434355432435454344

4444434543445443444434544

5555552334525232134545321

5213433224234553535212113

4344334353443433434434333

5545454354455445455544555

5434434545554434444543444

5555354335555553555354444

5232234421555543334344334

5434544445343553445544554

4453444332555554333534433

4444433444444444445333214

5545555554555555455444444

4554554534435345453344534

5454555555445555555545554

5555111111555511115123111

4433322322433333344333333

4454334443433445444545454

4455445544554454545445544

5555555545555555555555555

4344444422555533432343333

5555555545555544545554444

5544454555555554444445545

5534343343554543344344333

3335455444555555444444444

5555445444554555444444445

4345545433444545433444444

5555555555555555554355544

5444444444544444555444444

5555555555555555544555555

4544554445454533445445445

5444455444543334445554433

5544443544544544555544455

5454444444555554334454455

5555444443455555554444444

4455555553455553544354444

4334434431444444434433333

5552355353355534545444444

3332325322555552324555555

5544345442544554544355444

5532213341555543433344224

3435344433555543555355333

3435344433555544555355333

5354555454543334551223455

1234444445555554554544444

5555545545545545445544444

4444433323445433333333333

3444333333333334333333333

4343434343434343434343434

3333333355543334224544433

4444443453453455354354354

3434444444544543453344553

1223412444522345434245215

5445444454555554554444444

4345534522544532334534434

4343334444344444444444444

5444444454444444445444544

5555545555455555554455555

5554545455545455555555555

3444444444444443334344343

3334445545454533544322232

3444444453454544434434443

5545345434534444452344554

5454345354354444555435543

5435434451324234322324535

5435455454355445454554555

5444434444444444444444444

3343334432343433334333333

4445554334553555334344335

4554544444434544345344445

4444445545545555444344445

3434343434343434343434343

5555554553555545344344533

4444444443455544344344444

5454445555445555545555545

4544543433455455334344333

4543445443344444444444444

3444444444444443334344333

5455545455545555555554445

5445444455555545544444554

4453454445445445444543444

5454545455555555455454555

4454544544454545454545455

5555455554555555555555455

4444555554445444454555444

5544544544454445454445555

3333332333333333332233333

4444444444344444444444444

3444444444444444444444444

3434443334443334434254224

3344333433334343434333434

4555544443444444344444444

5555555535555555555555555

5554454444555555555555555

3444444434444444545444445

4555554544555554444455545

5555455554555555544455555

4444555555544444444555555

3333324232332332222323332

4444434532444345324433434

4455445445454555444455455

4444444444444435354334445

4555555555555555555555555

4555555544445555553332255

4545555555555555555555555

5445455555555454545554444

3333334444433444455555555

4444444343444444443334434

4454444454545455544344434

5444444445455555535554433

3211231222133121225544554

4544444455444454444445455

3444344434343333233333343

4555555454445554444555554

3333333333333331333333333

3434343434343343323133333

5444444443455544344444443

5544455453454545554555445

2333544444444444444444435

4455544535555555444443333

5555555555555555555455555

4344443443444444444344444

5554433444555555433355555

5534554555344445154545335

5553344553455545335343445

4553543543454545423454345

4443323333323333233232333

3344433333343344444333443

5555555555544534454544535

4455455554545454455555554

5444333444445555443334455

5444444444443335554433333

4445444545444354333342434

5544455333445553455443333

3444444444433434334444444

4444444444444444444444444

3444444444444444433444444

3333333333444333333333333

5545454544555555554435555

5555454555445445555455545

5555555445445455554555455

4555555544555555555445555

4445445444555444445555544

5554555554444555555444444

4445545545555555455455333

3445545555554444444455555

4444344444445444444444323

3443555554454555444444455

4434444354444444444544444

4453435535555544224244333

4554454552444555334344455

4444444444444444444333333

5544555555545554554444444

5554554555444344434444444

3333333343455444444444333

4444344433344444344444444

5545455554445555545544344

5555544555544454545454544

4345544344344344443444344

4444444443435443445433444

5411531515355115513314315

2312321221222222122223112

5443444454444554343333333

4444444443433444343344334

5443434444444453433434333

5455553553335443333354333

4445553344444554343344334

5444343444444544333444333

5554443353455544553333334

4345334232434445454433443

4444455454444454445444445

3243133341342534122223332

3243133341342534122223332

1555525555255154555555555

5555555555555555455344444

5555455555455545554455445

4445445444445444444444444

5444343355443544454335434

5555555554544555555455544

2535545522455553545344435

5555555554555555545455445

4445545534555554545455445

5554544433444454344345344

5455544544545455454345344

4444444544444444454345344

4333434344343433242125434

3333333333443335543444335

5445555543445554444455434

3445555543454555444455444

5555455535555553554445445

5555555543555555544455555

3233322334332343332343432

3433434434443444343344434

4444444454455534444444444

4353444353454445433344553

5553545551445543543455434

4353444355443544354535455

3453344445555433443543454

5544455544445454453455545

3333333334434343443434343

4545455544454545434443545

5555555545555555455555555

4545445545334544545554333

5545355531555555353345335

3333333443343433333233323

5554454545454454334445544

4333332331233432223344343

4444444444444444444433444

5554554554554545544555555

4444444443455543334244445

4444544444444434455544444

4444434433444445555545555

3334444445555434234422223

3233332333232434333334443

4333334342233532323333333

4223223443233323224334433

3334342553333433445233333

4444434444344544334334434

5444445444444544444444444

3344333454445444333433343

4444444434444444444444444

4444443444444443333344444

5554545534444543445344445

4444444444344443344434444

4334433544445534444433333

3333333443334533344434343

3334345554444234544343443

4444333435444324444444444

4344432444133432222233333

5455555455554555555555554

5555554544545544555554545

4443345554444434445543455

3334434424333343332123334

4445334335544545343343434

4333334443444434433324324

4243324344432424344434334

4555554554555455555554445

5455555555455355555555535

5555555554555554444555555

4555454335555544454544555

4333232233333323333233222

3453434433444434354544444

5445444413555534434434444

5555554443555555554444433

3445344352445545434434433

3444333343434434433334334

3445433432345545444554445

4444543443444445444454444

5555444453555545444444335

4555555545555544454434435

3444344342445555554333333

4555453225555554354545244

5445444555545555444544444

4444344444444434444344444

2112211232121132213222121

4345435554444544444444445

3445453345445545554344333

5545444454545545545445555

4555455434555555454534435

3454443354545444534345454

5444443454555545445445534

4555344455555545455445555

4334444443555454434324333

5434454443455554335444334

4444444444444444445544434

4545544445545445454444544

4555454545444555545454455

5555555445544555455554555

5555454434445554455455555

4544533434444455543344445

3444442444444545443223322

4545455544334443533543344

4445534323444545545444445

4554444554555554443454444

4444335534444343344434544

5555454455545455554344454

5545335355554453455344555

4345544443444454444445445

4555554455555555555555555

3455544455454544544544545

3555555544353355555543445

5554445544555554444344344

5554445544555554444344344

4444554445455554434333434

4444444455433444342233444

4534554555454555454545554

3443545455534555545454535

4553534555345455554355545

3454445435444534555545455

5435535444554355555455555

4434545453454455554355545

4545554554545454544355555

5553454545454545344544545

4555555555455555554555545

5455554535445455555555555

5455433455544344555545455

4444444444444444443434445

4444344543344454454435445

5554545555455555555555555

2345432123453212321234543

4444433443344333334333222

5454445543445544554555444

2123334341322234343433333

5555555555555555444555444

3454553445435453454455354

4555435454545544344343555

5554545454545434355544544

5222334422343443223234332

5443454445455544243333434

4444433443334433344234434

4444334343443443434344324

5554545545555554455454455

4455443553444544453344334

3334324422354534324144344

4444443344444444342344444

4445455454444444543445445

3444444444544533435234334

5455444444555324443422444

4344445344322523532132424

2324444433244442234244444

5344443343344243444443333

4554342143444542342244212

3222222333133533243223223

5555445434444444444344434

4555555533555553545355355

4444434444333543425244444

4235545442545542225344244

4434345445444444425244333

4544545344454444345244445

4344434444433444444444434

4344334332454434423233544

5444344435434444444444555

4444443333444444444444444

5355555555533555355535434

5555555354444543344344333

5344435355445543435433445

4544444344444443344344434

4445444544455254444453445

4543443344434544443334444

4444433334444333334434334

3333343323444433333323323

4434424233445533335424224

3444445424455334425444334

5444444234355454325134324

5554444344444443344344444

3344434443444434444333434

4454534554555454122133322

3343333333234433232234333

4444343344455544444333333

4444342434444442233343333

2453245121445522324122222

4333424453353524334143334

4444335524333523444245424

4254435445555534445234544

4444545443443434434243334

4433434244443444444333333

3334333242233434534333333

4334334455554545334233323

4344335432444443544544344

5444444334444543244244345

4444444442444444344433434

4444434333555444434344444

4445544442444554444454444

4333224252444432343211111

4443344345555555544344544

4444444444444444444244234

3341134331243412112111111

5434334344333434442333434

3344443321223323343423322

5234442224454444324224334

3322324211212432222221111

3545553522455443334333334

5555555545555545555455435

5444545544545455444444433

5555454555555555555555555

5554443435555354433453335

5555554555555555555554545

4444544443444444334333444

5555535545555554555444555

5554555555555555555445355

5555443342555544433333233

4444444444344444444333334

5453355452555553434333334

5555555555355555455444444

5444444454545554445433333

5555555543555554334333333

4443444444444434444433333

5443544445555553233244222

5444444545444544344433334

5555555533555555454555445

5555454522555554445335324

5445455544545545544455555

4444343434334444444433333

5554555554555554545534344

5555554455455554544433444

5555555555555554535334334

5435455555555443445334344

5554545554555544555455445

5555555553555554355555444

4444444444444444444433333

3555555553455553545455445

3443332253453232124223224

5555555555555555554555555

3443454342444443234234334

1344443321334544535433225

2444543433444543344344433

2444444443443444444444544

4444544442444434444444554

5555555555555555555555554

5454445443455443444454445

4444444444444444444354354

5555555555555555554445555

5554544455555553345544444

5444454544454455545445444

5545545455545554545454545

5545555555455554555554545

5455455454544445554455444

5434454544443444444533333

3445545552455432553344525

3554555542444552455344545

3444434453445543444444444

5334534333555233455344444

4554555555555544545444444

3444444432555554345455555

5555555555555555554445223

4555353255544555343224323

5555343353455555443324334

4554443333554544434433333

4444323344444544442245334

3555553354555555533344333

5554343343334444443333344

4555453352555555434455534

5555553343545544434344334

5555444334444444333333333

4555555555555555555455555

4555454453555555554433333

5555553353555555555355335

5555555555555555552333344

4555453322544555532334333

2443322311443443233233324

5455554454445545443334334

5555555555555555555353221

5555352252444553553144242

2222233322233223555445555

4445455555554545545455555

5444555444554444554455544

5555444453554555455444554

5445434355443454345454433

5444544554554334555555435

5554545454445455555545545

5554454555555445444345434

5554454555555554554445545

5555554455555555455455555

5555555554555555554455444

5454344444555554434343435

4544454445454454454554445

5555554555555554555544334

5555555555555554455444444

5545445434444544343444434

5555554555545554554454555

5443342353555554532455345

5444444454444544555544455

4554454544555544454544545

5555454545545553555544334

4545454454555445554555445

3555455534544544343344333

4443344343334443433443433

4555554443444454334455444

3333322333233333443332334

4445555544444455544455444

3222244423322522333223423

1111113222111111211122222

4444455532334433334443334

2111231212112111123311213

3321322232211222233322232

4444444332343433233234444

2311213313111123121121111

1221322312312112332111233

2444444334444343224234444

1222223233322333332333232

1222123132321233323344444

3211213213332211111111111

3444433443444444333434333

3444433443444444333434333

1111223313222122112232222

1111112112111112212211111

5445444454544443555434545

5555444554334455444455544

5554443214444544333344334

4545555545555545555555455

4544345455544534545445455

4555545545545544554545554

5555454455555445455444344

5545555555455454555555455

4334333332344543334335545

5454344444555554434343435

5445445554555455544445444

4444344444434444445555455

5555555455555554454555555

5443455554545554555543345

5555444555555555444445445

5445443433434544434433343

5555455455455554554555445

5355535555445535335555235

5444554445444544445444544

5545443434545554444454445

5444445454445555544444334

4555544544455445555454455

5555444444444555555354444

3333333444444343433323234

4554454444555554434344333

4453344322333444442244434

5555445544444454433445334

5555554454555555553455544

3333344444555554444433333

5555555544555555555555445

3555555555355555445555544

5555555555555555454355445

5555555544555553544444333

4444444433444544443434343

4444444433455554534444555

4555454554555555444534434

5555444452444444221344333

5554444454444444444444454

5555555555555552455535555

4333344344444242443343324

5555555555444444444555444

FINISH
