# Supplementary material for: New roles of Lagrange multiplier method in generalizability theory: Inference of estimating the optimal sample size for teaching ability evaluation of college teachers
Source: PLoS One. 2024 Oct 17;19(10):e0307710. doi: 10.1371/journal.pone.0307710 (PMC11486427; doi:10.1371/journal.pone.0307710)
Supplement: S1 File — For (s:t) ×i design, the (s: t)×i.doc is the program file in which contains data and the (s: t)×i_output.doc is result file. For (s:t) × (i:v) design, the (s: t)×(i: v).doc is the program file in which contains data and the (s: t) ×(i: v) _output.doc is result file. For (s:t) × (i:v) ×o design, the (s: t) ×(i: v)×o.doc is the program file in which contains data and the (s: t)×(i: v)×o_output.doc is result file. (ZIP) [file pone.0307710.s001.zip › 2024-8-19 supporting information files/2024-8-19 supporting information files/(sú║t)x(iú║v)xo_output.docx]

CONTROL CARDS FOR RUN 1

Control Cards File Name: 4.crd

(s:t)x(i:v)xo Design

GSTUDY (s:t)x(i:v)xo Design

OPTIONS NREC 5 "*.out" EMS SECI .8 NOBANNER TIME

EFFECT o 2

EFFECT t 19

EFFECT * s:t 22 25 25 31 60 19 25 29 35 17 22 64 27 26 20 21 21 22 19

EFFECT v 5

EFFECT i:v 5 5 5 5 5

FORMAT 0 1

PROCESS

INPUT RECORDS FOR RUN 1

(s:t)x(i:v)xo Design

RECORD NUMBER 1:

4.000 5.000 5.000 5.000 5.000 4.000 5.000 4.000 4.000 4.000

4.000 5.000 5.000 5.000 5.000 5.000 5.000 5.000 4.000 4.000

5.000 4.000 4.000 4.000 5.000

RECORD NUMBER 2:

5.000 5.000 5.000 4.000 5.000 4.000 5.000 3.000 5.000 5.000

5.000 5.000 5.000 5.000 4.000 5.000 5.000 5.000 5.000 4.000

5.000 5.000 5.000 5.000 4.000

RECORD NUMBER 3:

4.000 4.000 4.000 4.000 4.000 4.000 4.000 4.000 4.000 3.000

3.000 4.000 3.000 4.000 3.000 3.000 3.000 4.000 3.000 4.000

3.000 3.000 4.000 4.000 4.000

RECORD NUMBER 4:

4.000 5.000 5.000 4.000 5.000 5.000 5.000 5.000 4.000 5.000

5.000 5.000 5.000 4.000 5.000 5.000 5.000 5.000 4.000 5.000

4.000 5.000 5.000 5.000 5.000

RECORD NUMBER 5:

4.000 5.000 4.000 5.000 5.000 5.000 4.000 5.000 5.000 5.000

5.000 5.000 4.000 4.000 5.000 4.000 4.000 5.000 5.000 5.000

4.000 5.000 5.000 5.000 5.000

MEANS FOR MAIN EFFECTS FOR RUN 1

(s:t)x(i:v)xo Design

Means for o

3.906 3.993

Means for t

4.065 4.200 3.996 4.262 3.986 3.374 4.062 3.622 4.101 4.039

4.292 3.603 4.176 4.006 3.865 4.341 3.004 4.156 4.211

Means for s:t

4.060 4.500 3.600 4.300 4.320 3.940 3.320 3.920 4.320 4.380

3.860 4.160 3.740 4.260 4.160 4.540 4.220 4.740 3.600 3.340

3.860 4.280 4.280 3.600 4.280 4.480 4.260 4.540 4.520 4.300

4.800 4.200 4.500 4.320 4.300 4.160 4.300 4.280 4.440 3.600

4.180 4.180 3.800 3.600 4.020 3.640 4.420 3.980 4.440 3.720

3.900 4.160 4.160 4.180 4.000 3.520 4.160 3.380 3.420 3.900

4.640 4.840 3.940 4.280 4.060 4.240 3.920 3.360 4.480 3.600

3.320 4.300 3.960 4.400 3.960 4.500 4.100 4.600 4.340 3.860

3.560 4.340 4.560 4.200 4.300 4.480 4.740 4.040 4.500 3.920

4.440 4.400 4.140 3.920 4.400 4.940 4.320 3.960 4.760 4.820

3.940 3.660 4.060 4.500 4.260 4.260 4.420 4.600 4.400 4.320

3.920 4.360 4.360 3.620 4.480 2.520 4.300 3.320 3.460 3.720

3.820 3.860 4.200 4.260 3.960 3.980 4.100 3.960 3.740 3.240

3.420 3.780 4.700 3.500 3.420 3.680 3.900 3.940 3.860 3.960

3.440 3.940 4.240 4.060 4.520 3.940 4.420 4.360 3.860 4.220

4.320 4.440 2.760 4.260 4.020 4.540 3.580 3.880 4.100 4.300

4.300 3.880 3.680 2.540 3.060 2.880 2.760 3.020 3.760 3.060

3.340 3.740 3.480 3.780 2.960 2.900 4.380 4.260 3.040 4.040

2.940 4.160 3.640 4.380 3.960 4.460 4.220 3.960 3.460 3.220

3.840 4.160 4.180 4.420 3.020 3.780 4.420 4.320 4.520 3.980

3.980 4.480 3.920 3.740 4.700 4.540 4.260 3.220 3.520 3.380

3.880 4.140 3.680 3.960 3.460 3.280 3.420 3.380 3.920 4.020

3.700 3.400 3.600 3.760 4.040 4.260 3.400 3.380 3.660 2.660

4.100 3.700 3.740 3.800 3.860 2.720 4.060 3.160 4.020 4.640

4.620 4.400 4.500 3.020 3.720 4.060 4.260 3.900 4.120 4.180

4.400 4.360 4.040 4.300 4.360 4.300 4.280 2.640 4.360 4.020

4.320 4.600 4.160 4.440 4.100 3.740 4.160 3.860 3.800 4.360

4.260 4.440 4.020 3.540 3.500 4.300 3.960 3.800 3.900 3.940

4.060 4.880 4.180 4.080 4.120 3.940 3.980 4.020 4.260 4.520

4.660 4.620 3.960 4.400 4.520 4.280 4.680 4.560 4.520 4.400

3.340 4.600 3.680 3.380 4.600 3.720 4.700 4.440 4.380 4.200

3.420 3.600 3.660 3.680 4.740 3.200 3.360 3.340 4.140 3.720

3.700 3.720 3.020 3.620 3.180 2.660 3.440 3.840 3.120 3.640

3.620 4.120 3.960 3.260 3.340 3.720 4.260 3.840 3.960 3.660

3.880 3.340 3.960 3.360 2.440 3.680 3.600 3.900 4.040 3.760

3.480 3.200 3.760 3.160 3.240 3.600 3.820 3.740 3.600 3.300

3.500 3.640 4.300 3.940 3.660 4.320 3.280 4.140 3.920 2.840

3.540 3.160 4.020 2.920 4.100 4.600 4.120 4.660 4.440 4.360

3.640 4.280 4.740 3.960 3.520 3.880 4.200 4.040 3.980 3.900

3.720 4.280 4.140 4.080 4.600 3.960 4.180 4.600 4.180 4.180

4.400 4.240 3.960 4.140 3.140 4.200 3.820 3.740 3.460 3.540

3.960 4.280 4.020 4.040 4.720 3.660 4.600 4.800 4.880 4.480

3.980 4.100 3.780 3.600 3.540 3.680 3.800 4.000 3.620 3.620

3.800 3.960 4.160 3.500 4.360 3.700 3.520 4.400 4.220 3.780

4.160 3.620 3.160 3.800 4.200 4.000 3.720 4.740 4.020 4.020

4.360 4.220 4.620 4.400 4.760 4.060 4.540 4.440 4.260 4.720

4.560 4.380 4.540 4.000 3.580 3.820 4.580 4.540 2.980 3.620

3.180 3.000 3.620 3.320 2.380 3.740 2.560 2.840 3.680 2.580

3.200 3.420 2.660 2.900 2.200 3.140 2.580 3.160 2.320 4.420

4.000 3.280 4.680 3.840 4.540 4.440 4.640 3.340 3.980 4.400

4.240 4.840 4.200 4.680 4.060 4.220 3.660 3.780 3.400 4.340

4.460 4.080 4.060 4.180 4.060 4.100 4.420 3.840 4.440 4.220

4.840 4.100 4.000 4.100 4.620 3.940 4.360 4.160 3.860 4.620

Means for v

4.055 3.919 4.152 3.803 3.819

Means for i:v

4.051 4.057 4.068 4.094 4.005 4.016 4.012 4.008 3.865 3.696

4.076 4.150 4.188 4.252 4.092 3.825 3.851 3.798 3.904 3.639

3.862 3.930 3.747 3.628 3.929

ANOVA TABLE FOR RUN 1

(s:t)x(i:v)xo Design

-----------------------------------------------------------------------------

Effect df T SS MS VC

-----------------------------------------------------------------------------

o 1 413461.55426 50.60242 50.60242 0.00147

t 18 416000.60543 2589.65358 143.86964 0.08300

s:t 511 420080.16000 4079.55457 7.98347 0.00288

v 4 413893.70415 482.75230 120.68808 0.02021

i:v 20 414117.65660 223.95245 11.19762 0.00850

ot 18 416532.90857 481.70073 26.76115 0.02704

os:t 511 424633.68000 4021.21686 7.86931 0.28058

ov 4 413950.35472 6.04815 1.51204 -0.00018

oi:v 20 414203.28679 28.97962 1.44898 0.00160

tv 72 416742.08231 258.72458 3.59340 0.00692

ti:v 360 417387.98544 421.95068 1.17209 0.01110

sv:t 2044 422508.20000 1686.56312 0.82513 -0.00224

si:tv 10220 427177.00000 4022.89687 0.39363 -0.00366

otv 72 417359.79579 79.36219 1.10225 0.00059

oti:v 360 418238.83898 204.16043 0.56711 0.00602

osv:t 2044 428894.40000 1747.26966 0.85483 0.09078

osi:tv 10220 437894.00000 4097.65994 0.40095 0.40095

-----------------------------------------------------------------------------

Mean 413410.95185

-----------------------------------------------------------------------------

Total 26499 24483.04815

-----------------------------------------------------------------------------

Grand Mean: 3.94974

STANDARD ERRORS AND CONFIDENCE INTERVALS FOR VARIANCE COMPONENTS FOR RUN 1

TING ET AL. INTERVALS

(s:t)x(i:v)xo Design

***Output not provided because design is unbalanced***

EXPECTED MEAN SQUARE EQUATIONS FOR RUN 1

(s:t)x(i:v)xo Design

EMS(o) = 1.000*VC(osi:tv) + 5.000*VC(osv:t) + 33.487*VC(oti:v)

+ 167.434*VC(otv) + 530.000*VC(oi:v)

+2650.000*VC(ov) + 25.000*VC(os:t)

+ 837.170*VC(ot) +13250.000*VC(o)

EMS(t) = 1.000*VC(osi:tv) + 5.000*VC(osv:t) + 27.584*VC(oti:v)

+ 137.920*VC(otv) + 2.000*VC(si:tv)

+ 10.000*VC(sv:t) + 55.168*VC(ti:v)

+ 275.841*VC(tv) + 25.000*VC(os:t)

+ 689.602*VC(ot) + 50.000*VC(s:t)

+1379.203*VC(t)

EMS(s:t) = 1.000*VC(osi:tv) + 5.000*VC(osv:t) + 2.000*VC(si:tv)

+ 10.000*VC(sv:t) + 25.000*VC(os:t)

+ 50.000*VC(s:t)

EMS(v) = 1.000*VC(osi:tv) + 5.000*VC(osv:t) + 33.487*VC(oti:v)

+ 167.434*VC(otv) + 2.000*VC(si:tv)

+ 10.000*VC(sv:t) + 66.974*VC(ti:v)

+ 334.868*VC(tv) + 530.000*VC(oi:v)

+2650.000*VC(ov) +1060.000*VC(i:v)

+5300.000*VC(v)

EMS(i:v) = 1.000*VC(osi:tv) + 33.487*VC(oti:v) + 2.000*VC(si:tv)

+ 66.974*VC(ti:v) + 530.000*VC(oi:v)

+1060.000*VC(i:v)

EMS(ot) = 1.000*VC(osi:tv) + 5.000*VC(osv:t) + 27.584*VC(oti:v)

+ 137.920*VC(otv) + 25.000*VC(os:t)

+ 689.602*VC(ot)

EMS(os:t) = 1.000*VC(osi:tv) + 5.000*VC(osv:t) + 25.000*VC(os:t)

EMS(ov) = 1.000*VC(osi:tv) + 5.000*VC(osv:t) + 33.487*VC(oti:v)

+ 167.434*VC(otv) + 530.000*VC(oi:v)

+2650.000*VC(ov)

EMS(oi:v) = 1.000*VC(osi:tv) + 33.487*VC(oti:v) + 530.000*VC(oi:v)

EMS(tv) = 1.000*VC(osi:tv) + 5.000*VC(osv:t) + 27.584*VC(oti:v)

+ 137.920*VC(otv) + 2.000*VC(si:tv)

+ 10.000*VC(sv:t) + 55.168*VC(ti:v)

+ 275.841*VC(tv)

EMS(ti:v) = 1.000*VC(osi:tv) + 27.584*VC(oti:v) + 2.000*VC(si:tv)

+ 55.168*VC(ti:v)

EMS(sv:t) = 1.000*VC(osi:tv) + 5.000*VC(osv:t) + 2.000*VC(si:tv)

+ 10.000*VC(sv:t)

EMS(si:tv) = 1.000*VC(osi:tv) + 2.000*VC(si:tv)

EMS(otv) = 1.000*VC(osi:tv) + 5.000*VC(osv:t) + 27.584*VC(oti:v)

+ 137.920*VC(otv)

EMS(oti:v) = 1.000*VC(osi:tv) + 27.584*VC(oti:v)

EMS(osv:t) = 1.000*VC(osi:tv) + 5.000*VC(osv:t)

EMS(osi:tv) = 1.000*VC(osi:tv)

*** EMS matrix is upper diagonal***

Date and time at beginning of Run 1: Sun Jan 8 16:35:13 2017

Processor time for run: 1 seconds
