# Supplementary material for: New roles of Lagrange multiplier method in generalizability theory: Inference of estimating the optimal sample size for teaching ability evaluation of college teachers
Source: PLoS One. 2024 Oct 17;19(10):e0307710. doi: 10.1371/journal.pone.0307710 (PMC11486427; doi:10.1371/journal.pone.0307710)
Supplement: S1 File — For (s:t) ×i design, the (s: t)×i.doc is the program file in which contains data and the (s: t)×i_output.doc is result file. For (s:t) × (i:v) design, the (s: t)×(i: v).doc is the program file in which contains data and the (s: t) ×(i: v) _output.doc is result file. For (s:t) × (i:v) ×o design, the (s: t) ×(i: v)×o.doc is the program file in which contains data and the (s: t)×(i: v)×o_output.doc is result file. (ZIP) [file pone.0307710.s001.zip › 2024-8-19 supporting information files/2024-8-19 supporting information files/(sú║t)xi_output.docx]

****************************************************************************************

****************************************************************************************

GGGGGGG EEEEEEEE N N OOOOO V V AAA

G G E N N N O O V V A A

U U RRRR G E N N N O O V V A A

U U R R G EEEEE N N N O O V V AAAAAAAAA

U U RRRR G GGG E N N N O O V V A A

U U R R G G E N N N O O V V A A

UUU R R GGGGGGG EEEEEEEE N N OOOOO V A A

Version 2.1 Robert L. Brennan

June 2001 Iowa Testing Programs

All Rights Reserved University of Iowa

****************************************************************************************

****************************************************************************************

CONTROL CARDS FOR RUN 1

Control Cards File Name: 2.crd

(s:t)xi Design

GSTUDY (s:t)xi Design

OPTIONS NREC 5 "*.out" EMS TIME

EFFECT * t 19

EFFECT s:t 22 25 25 31 60 19 25 29 35 17 22 64 27 26 20 21 21 22 19

EFFECT i 25

FORMAT 0 1

PROCESS

INPUT RECORDS FOR RUN 1

(s:t)xi Design

RECORD NUMBER 1:

4.000 4.000 3.000 4.000 3.000 3.000 4.000 4.000 3.000 4.000

4.000 4.000 3.000 4.000 3.000 3.000 4.000 4.000 4.000 3.000

3.000 4.000 3.000 3.000 4.000 5.000 5.000 5.000 5.000 5.000

4.000 4.000 5.000 3.000 4.000 5.000 5.000 5.000 5.000 5.000

3.000 4.000 3.000 5.000 3.000 5.000 4.000 3.000 3.000 4.000

4.000 4.000 5.000 4.000 3.000 3.000 3.000 3.000 3.000 3.000

4.000 4.000 4.000 4.000 4.000 3.000 3.000 3.000 4.000 3.000

4.000 4.000 3.000 3.000 4.000 3.000 5.000 4.000 3.000 5.000

4.000 3.000 4.000 3.000 4.000 3.000 5.000 5.000 4.000 3.000

2.000 4.000 3.000 5.000 4.000 5.000 4.000 3.000 4.000 4.000

4.000 4.000 4.000 4.000 4.000 3.000 4.000 5.000 4.000 3.000

4.000 4.000 5.000 4.000 4.000 3.000 4.000 4.000 4.000 4.000

3.000 4.000 5.000 4.000 4.000 5.000 5.000 5.000 5.000 5.000

5.000 2.000 3.000 3.000 4.000 5.000 2.000 5.000 2.000 3.000

2.000 1.000 3.000 4.000 5.000 4.000 5.000 3.000 2.000 1.000

5.000 2.000 1.000 3.000 4.000 3.000 3.000 2.000 2.000 4.000

2.000 3.000 4.000 5.000 5.000 3.000 5.000 3.000 5.000 2.000

1.000 2.000 1.000 1.000 3.000 4.000 3.000 4.000 4.000 3.000

3.000 4.000 3.000 5.000 3.000 4.000 4.000 3.000 4.000 3.000

3.000 4.000 3.000 4.000 4.000 3.000 4.000 3.000 3.000 3.000

5.000 5.000 4.000 5.000 4.000 5.000 4.000 3.000 5.000 4.000

4.000 5.000 5.000 4.000 4.000 5.000 4.000 5.000 5.000 5.000

4.000 4.000 5.000 5.000 5.000 5.000 4.000 3.000 4.000 4.000

3.000 4.000 5.000 4.000 5.000 5.000 5.000 4.000 4.000 3.000

4.000 4.000 4.000 4.000 5.000 4.000 3.000 4.000 4.000 4.000

5.000 5.000 5.000 5.000 3.000 5.000 4.000 3.000 3.000 5.000

5.000 5.000 5.000 5.000 5.000 3.000 5.000 5.000 5.000 3.000

5.000 4.000 4.000 4.000 4.000 5.000 2.000 3.000 2.000 2.000

3.000 4.000 4.000 2.000 1.000 5.000 5.000 5.000 5.000 4.000

3.000 3.000 3.000 4.000 3.000 4.000 4.000 3.000 3.000 4.000

5.000 4.000 3.000 4.000 5.000 4.000 4.000 4.000 4.000 5.000

3.000 4.000 3.000 5.000 5.000 3.000 4.000 4.000 5.000 5.000

4.000 4.000 5.000 5.000 4.000 4.000 4.000 5.000 3.000 4.000

4.000 4.000 3.000 3.000 2.000 5.000 5.000 5.000 5.000 5.000

4.000 3.000 3.000 3.000 5.000 3.000 4.000 4.000 3.000 3.000

4.000 4.000 4.000 4.000 4.000 3.000 3.000 4.000 4.000 4.000

4.000 4.000 4.000 4.000 4.000 4.000 4.000 4.000 5.000 3.000

3.000 3.000 2.000 1.000 4.000 5.000 5.000 4.000 5.000 5.000

5.000 5.000 5.000 5.000 4.000 5.000 5.000 5.000 5.000 5.000

5.000 4.000 5.000 5.000 4.000 4.000 4.000 4.000 4.000 4.000

4.000 5.000 5.000 4.000 5.000 5.000 4.000 5.000 3.000 4.000

4.000 3.000 5.000 3.000 4.000 5.000 4.000 5.000 3.000 3.000

4.000 4.000 5.000 3.000 4.000 5.000 4.000 5.000 4.000 5.000

5.000 5.000 5.000 5.000 5.000 4.000 4.000 5.000 5.000 5.000

5.000 5.000 5.000 5.000 5.000 4.000 5.000 5.000 5.000 4.000

5.000 5.000 5.000 5.000 1.000 1.000 1.000 1.000 1.000 1.000

5.000 5.000 5.000 5.000 1.000 1.000 1.000 1.000 5.000 1.000

2.000 3.000 1.000 1.000 1.000 4.000 4.000 3.000 3.000 3.000

2.000 2.000 3.000 2.000 2.000 4.000 3.000 3.000 3.000 3.000

3.000 3.000 4.000 4.000 3.000 3.000 3.000 3.000 3.000 3.000

4.000 4.000 5.000 4.000 3.000 3.000 4.000 4.000 4.000 3.000

4.000 3.000 3.000 4.000 4.000 5.000 4.000 4.000 4.000 5.000

4.000 5.000 4.000 5.000 4.000 4.000 4.000 5.000 5.000 4.000

4.000 5.000 5.000 4.000 4.000 5.000 5.000 4.000 4.000 5.000

4.000 5.000 4.000 5.000 4.000 4.000 5.000 5.000 4.000 4.000

RECORD NUMBER 2:

5.000 5.000 5.000 5.000 5.000 5.000 5.000 5.000 4.000 5.000

5.000 5.000 5.000 5.000 5.000 5.000 5.000 5.000 5.000 5.000

5.000 5.000 5.000 5.000 5.000 4.000 3.000 4.000 4.000 4.000

4.000 4.000 4.000 2.000 2.000 5.000 5.000 5.000 5.000 3.000

3.000 4.000 3.000 2.000 3.000 4.000 3.000 3.000 3.000 3.000

5.000 5.000 5.000 5.000 5.000 5.000 5.000 5.000 4.000 5.000

5.000 5.000 5.000 5.000 4.000 4.000 5.000 4.000 5.000 5.000

5.000 4.000 4.000 4.000 4.000 5.000 5.000 4.000 4.000 4.000

5.000 4.000 5.000 5.000 5.000 5.000 5.000 5.000 5.000 5.000

4.000 4.000 4.000 4.000 4.000 4.000 5.000 5.000 4.000 5.000

5.000 5.000 3.000 4.000 3.000 4.000 3.000 3.000 4.000 3.000

5.000 5.000 4.000 5.000 4.000 3.000 3.000 4.000 4.000 3.000

4.000 4.000 3.000 3.000 3.000 3.000 3.000 3.000 5.000 4.000

5.000 5.000 4.000 4.000 4.000 5.000 5.000 5.000 5.000 5.000

5.000 4.000 4.000 4.000 4.000 4.000 4.000 4.000 4.000 4.000

5.000 5.000 5.000 5.000 4.000 4.000 5.000 4.000 4.000 4.000

5.000 5.000 4.000 5.000 5.000 5.000 4.000 4.000 4.000 4.000

4.000 4.000 4.000 4.000 5.000 4.000 3.000 4.000 5.000 5.000

4.000 5.000 4.000 3.000 3.000 4.000 4.000 4.000 5.000 4.000

5.000 4.000 3.000 3.000 4.000 4.000 4.000 4.000 4.000 4.000

5.000 5.000 5.000 5.000 5.000 5.000 5.000 5.000 5.000 5.000

5.000 5.000 5.000 5.000 5.000 5.000 5.000 5.000 4.000 3.000

5.000 5.000 5.000 4.000 4.000 5.000 4.000 4.000 4.000 4.000

4.000 4.000 4.000 4.000 4.000 5.000 4.000 4.000 4.000 4.000

4.000 5.000 5.000 5.000 4.000 4.000 4.000 4.000 4.000 4.000

5.000 5.000 5.000 5.000 5.000 5.000 5.000 5.000 5.000 5.000

5.000 5.000 5.000 5.000 5.000 5.000 5.000 4.000 4.000 5.000

5.000 5.000 5.000 5.000 5.000 4.000 5.000 4.000 4.000 5.000

5.000 4.000 4.000 4.000 5.000 4.000 5.000 4.000 5.000 3.000

3.000 4.000 4.000 5.000 4.000 4.000 5.000 4.000 4.000 5.000

5.000 4.000 4.000 4.000 4.000 5.000 5.000 4.000 4.000 4.000

5.000 4.000 3.000 3.000 3.000 4.000 4.000 4.000 5.000 5.000

5.000 4.000 4.000 3.000 3.000 5.000 5.000 4.000 4.000 4.000

4.000 3.000 5.000 4.000 4.000 5.000 4.000 4.000 5.000 4.000

4.000 5.000 5.000 5.000 5.000 4.000 4.000 4.000 5.000 5.000

5.000 4.000 5.000 4.000 4.000 4.000 4.000 4.000 4.000 4.000

5.000 5.000 5.000 5.000 5.000 4.000 3.000 3.000 4.000 4.000

5.000 4.000 4.000 5.000 5.000 5.000 5.000 5.000 5.000 4.000

4.000 4.000 4.000 4.000 3.000 4.000 5.000 5.000 5.000 5.000

5.000 5.000 5.000 4.000 4.000 4.000 4.000 4.000 4.000 4.000

4.000 4.000 5.000 5.000 5.000 5.000 5.000 5.000 5.000 3.000

4.000 5.000 5.000 5.000 5.000 3.000 5.000 4.000 4.000 3.000

5.000 4.000 4.000 4.000 4.000 4.000 3.000 3.000 4.000 4.000

3.000 4.000 4.000 3.000 1.000 4.000 4.000 4.000 4.000 4.000

4.000 4.000 3.000 4.000 4.000 3.000 3.000 3.000 3.000 3.000

5.000 5.000 5.000 2.000 3.000 5.000 5.000 3.000 5.000 3.000

3.000 5.000 5.000 5.000 3.000 4.000 5.000 4.000 5.000 4.000

4.000 4.000 4.000 4.000 4.000 3.000 3.000 3.000 2.000 3.000

2.000 5.000 3.000 2.000 2.000 5.000 5.000 5.000 5.000 5.000

2.000 3.000 2.000 4.000 5.000 5.000 5.000 5.000 5.000 5.000

5.000 5.000 4.000 4.000 3.000 4.000 5.000 4.000 4.000 2.000

5.000 4.000 4.000 5.000 5.000 4.000 5.000 4.000 4.000 3.000

5.000 5.000 4.000 4.000 4.000 5.000 5.000 3.000 2.000 2.000

1.000 3.000 3.000 4.000 1.000 5.000 5.000 5.000 5.000 4.000

3.000 4.000 3.000 3.000 3.000 4.000 4.000 2.000 2.000 4.000

3.000 4.000 3.000 5.000 3.000 4.000 4.000 4.000 3.000 3.000

5.000 5.000 5.000 5.000 4.000 3.000 5.000 5.000 5.000 3.000

5.000 5.000 3.000 3.000 3.000 3.000 4.000 3.000 5.000 3.000

4.000 4.000 4.000 3.000 3.000 5.000 5.000 5.000 5.000 4.000

4.000 5.000 5.000 5.000 3.000 5.000 5.000 3.000 3.000 3.000

5.000 3.000 5.000 4.000 5.000 5.000 5.000 4.000 5.000 4.000

5.000 4.000 3.000 3.000 3.000 4.000 5.000 5.000 1.000 2.000

2.000 3.000 4.000 5.000 5.000

RECORD NUMBER 3:

1.000 2.000 3.000 4.000 4.000 4.000 4.000 4.000 4.000 5.000

5.000 5.000 5.000 5.000 5.000 4.000 5.000 5.000 4.000 5.000

4.000 4.000 4.000 4.000 4.000 5.000 5.000 5.000 5.000 5.000

4.000 5.000 5.000 4.000 5.000 5.000 4.000 5.000 5.000 4.000

5.000 4.000 4.000 5.000 5.000 4.000 4.000 4.000 4.000 4.000

4.000 4.000 4.000 4.000 4.000 3.000 3.000 3.000 2.000 3.000

4.000 4.000 5.000 4.000 3.000 3.000 3.000 3.000 3.000 3.000

3.000 3.000 3.000 3.000 3.000 3.000 4.000 4.000 4.000 3.000

3.000 3.000 3.000 3.000 3.000 3.000 3.000 3.000 3.000 3.000

4.000 3.000 3.000 3.000 3.000 3.000 3.000 3.000 3.000 3.000

4.000 3.000 4.000 3.000 4.000 3.000 4.000 3.000 4.000 3.000

4.000 3.000 4.000 3.000 4.000 3.000 4.000 3.000 4.000 3.000

4.000 3.000 4.000 3.000 4.000 3.000 3.000 3.000 3.000 3.000

3.000 3.000 3.000 5.000 5.000 5.000 4.000 3.000 3.000 3.000

4.000 2.000 2.000 4.000 5.000 4.000 4.000 4.000 3.000 3.000

4.000 4.000 4.000 4.000 4.000 4.000 3.000 4.000 5.000 3.000

4.000 5.000 3.000 4.000 5.000 5.000 3.000 5.000 4.000 3.000

5.000 4.000 3.000 5.000 4.000 3.000 4.000 3.000 4.000 4.000

4.000 4.000 4.000 4.000 4.000 5.000 4.000 4.000 5.000 4.000

3.000 4.000 5.000 3.000 3.000 4.000 4.000 5.000 5.000 3.000

1.000 2.000 2.000 3.000 4.000 1.000 2.000 4.000 4.000 4.000

5.000 2.000 2.000 3.000 4.000 5.000 4.000 3.000 4.000 2.000

4.000 5.000 2.000 1.000 5.000 5.000 4.000 4.000 5.000 4.000

4.000 4.000 4.000 5.000 4.000 5.000 5.000 5.000 5.000 5.000

4.000 5.000 5.000 4.000 4.000 4.000 4.000 4.000 4.000 4.000

4.000 3.000 4.000 5.000 5.000 3.000 4.000 5.000 2.000 2.000

5.000 4.000 4.000 5.000 3.000 2.000 3.000 3.000 4.000 5.000

3.000 4.000 4.000 3.000 4.000 4.000 3.000 4.000 3.000 3.000

3.000 4.000 4.000 4.000 4.000 3.000 4.000 4.000 4.000 4.000

4.000 4.000 4.000 4.000 4.000 4.000 4.000 4.000 4.000 4.000

5.000 4.000 4.000 4.000 4.000 4.000 4.000 4.000 5.000 4.000

4.000 4.000 4.000 4.000 4.000 4.000 4.000 4.000 5.000 4.000

4.000 4.000 5.000 4.000 4.000 5.000 5.000 5.000 5.000 5.000

4.000 5.000 5.000 5.000 5.000 4.000 5.000 5.000 5.000 5.000

5.000 5.000 5.000 4.000 4.000 5.000 5.000 5.000 5.000 5.000

5.000 5.000 5.000 4.000 5.000 4.000 5.000 4.000 5.000 5.000

5.000 4.000 5.000 4.000 5.000 5.000 5.000 5.000 5.000 5.000

5.000 5.000 5.000 5.000 5.000 3.000 4.000 4.000 4.000 4.000

4.000 4.000 4.000 4.000 4.000 4.000 4.000 4.000 4.000 4.000

3.000 3.000 3.000 4.000 3.000 4.000 4.000 3.000 4.000 3.000

3.000 3.000 3.000 4.000 4.000 4.000 5.000 5.000 4.000 5.000

4.000 5.000 4.000 5.000 3.000 3.000 5.000 4.000 4.000 3.000

2.000 2.000 2.000 3.000 2.000 3.000 4.000 4.000 4.000 4.000

4.000 4.000 4.000 5.000 3.000 4.000 5.000 4.000 5.000 4.000

4.000 4.000 3.000 4.000 4.000 3.000 4.000 4.000 4.000 3.000

5.000 5.000 4.000 5.000 3.000 4.000 5.000 4.000 3.000 4.000

5.000 3.000 4.000 4.000 4.000 4.000 4.000 5.000 2.000 3.000

4.000 4.000 5.000 5.000 4.000 5.000 4.000 5.000 4.000 3.000

4.000 5.000 3.000 5.000 4.000 3.000 5.000 4.000 4.000 4.000

4.000 5.000 5.000 5.000 4.000 3.000 5.000 5.000 4.000 3.000

5.000 4.000 3.000 5.000 4.000 3.000 4.000 4.000 5.000 1.000

3.000 2.000 4.000 2.000 3.000 4.000 3.000 2.000 2.000 3.000

2.000 4.000 5.000 3.000 5.000 5.000 4.000 3.000 5.000 4.000

5.000 5.000 4.000 5.000 4.000 3.000 5.000 5.000 4.000 4.000

5.000 4.000 5.000 4.000 5.000 5.000 4.000 5.000 5.000 5.000

5.000 4.000 4.000 4.000 4.000 3.000 4.000 4.000 4.000 4.000

4.000 4.000 4.000 4.000 4.000 4.000 4.000 4.000 4.000 4.000

4.000 4.000 4.000 4.000 4.000 3.000 3.000 4.000 3.000 3.000

3.000 4.000 4.000 3.000 2.000 3.000 4.000 3.000 4.000 3.000

3.000 3.000 3.000 4.000 3.000 3.000 3.000 3.000 3.000 3.000

4.000 4.000 4.000 5.000 5.000 5.000 4.000 3.000 3.000 4.000

5.000 5.000 3.000 5.000 5.000 5.000 3.000 3.000 4.000 3.000

4.000 4.000 3.000 3.000 5.000

RECORD NUMBER 4:

4.000 5.000 5.000 4.000 5.000 4.000 4.000 4.000 4.000 4.000

4.000 3.000 4.000 5.000 4.000 4.000 3.000 4.000 5.000 3.000

4.000 4.000 4.000 4.000 5.000 4.000 4.000 4.000 4.000 4.000

4.000 5.000 5.000 4.000 5.000 5.000 4.000 5.000 5.000 5.000

5.000 4.000 4.000 4.000 3.000 4.000 4.000 4.000 4.000 5.000

3.000 4.000 3.000 4.000 3.000 4.000 3.000 4.000 3.000 4.000

3.000 4.000 3.000 4.000 3.000 4.000 3.000 4.000 3.000 4.000

3.000 4.000 3.000 4.000 3.000 5.000 5.000 5.000 5.000 5.000

5.000 4.000 5.000 5.000 3.000 5.000 5.000 5.000 5.000 4.000

5.000 3.000 4.000 4.000 3.000 4.000 4.000 5.000 3.000 3.000

4.000 4.000 4.000 4.000 4.000 4.000 4.000 4.000 4.000 3.000

4.000 5.000 5.000 5.000 4.000 4.000 3.000 4.000 4.000 3.000

4.000 4.000 4.000 4.000 4.000 5.000 4.000 5.000 4.000 4.000

4.000 5.000 5.000 5.000 5.000 4.000 4.000 5.000 5.000 5.000

5.000 5.000 4.000 5.000 5.000 5.000 5.000 5.000 4.000 5.000

4.000 5.000 4.000 4.000 5.000 4.000 3.000 4.000 3.000 3.000

4.000 5.000 5.000 4.000 5.000 5.000 3.000 3.000 4.000 3.000

4.000 4.000 3.000 3.000 3.000 4.000 5.000 4.000 3.000 4.000

4.000 5.000 4.000 4.000 3.000 3.000 4.000 4.000 4.000 4.000

4.000 4.000 4.000 4.000 4.000 4.000 4.000 4.000 4.000 4.000

3.000 4.000 4.000 4.000 4.000 4.000 4.000 4.000 4.000 4.000

4.000 4.000 4.000 4.000 4.000 3.000 3.000 3.000 4.000 3.000

4.000 4.000 3.000 3.000 3.000 5.000 4.000 5.000 5.000 5.000

4.000 5.000 4.000 5.000 5.000 5.000 4.000 5.000 5.000 5.000

5.000 5.000 5.000 5.000 5.000 5.000 4.000 4.000 4.000 5.000

5.000 4.000 4.000 5.000 4.000 4.000 4.000 4.000 5.000 5.000

5.000 5.000 5.000 5.000 4.000 5.000 5.000 4.000 4.000 4.000

4.000 4.000 5.000 5.000 4.000 4.000 4.000 5.000 3.000 4.000

5.000 4.000 4.000 4.000 5.000 4.000 4.000 5.000 4.000 4.000

5.000 4.000 4.000 4.000 5.000 4.000 3.000 4.000 4.000 4.000

5.000 4.000 5.000 4.000 5.000 4.000 5.000 4.000 5.000 5.000

5.000 5.000 5.000 5.000 5.000 5.000 4.000 5.000 5.000 4.000

5.000 4.000 5.000 5.000 5.000 4.000 4.000 5.000 4.000 5.000

4.000 4.000 5.000 4.000 4.000 4.000 5.000 4.000 5.000 4.000

5.000 4.000 5.000 4.000 5.000 4.000 5.000 4.000 5.000 5.000

5.000 5.000 5.000 5.000 4.000 5.000 5.000 5.000 5.000 4.000

5.000 5.000 5.000 5.000 5.000 5.000 5.000 5.000 5.000 5.000

5.000 5.000 4.000 5.000 5.000 4.000 4.000 4.000 4.000 5.000

5.000 5.000 5.000 5.000 4.000 4.000 4.000 5.000 4.000 4.000

4.000 4.000 5.000 4.000 5.000 5.000 5.000 4.000 4.000 4.000

5.000 5.000 4.000 4.000 5.000 4.000 4.000 5.000 4.000 4.000

4.000 5.000 4.000 4.000 4.000 5.000 4.000 5.000 4.000 4.000

4.000 5.000 5.000 5.000 5.000 3.000 3.000 3.000 3.000 3.000

3.000 2.000 3.000 3.000 3.000 3.000 3.000 3.000 3.000 3.000

3.000 3.000 3.000 2.000 2.000 3.000 3.000 3.000 3.000 3.000

4.000 4.000 4.000 4.000 4.000 4.000 4.000 4.000 4.000 4.000

3.000 4.000 4.000 4.000 4.000 4.000 4.000 4.000 4.000 4.000

4.000 4.000 4.000 4.000 4.000 3.000 4.000 4.000 4.000 4.000

4.000 4.000 4.000 4.000 4.000 4.000 4.000 4.000 4.000 4.000

4.000 4.000 4.000 4.000 4.000 4.000 4.000 4.000 4.000 4.000

3.000 4.000 3.000 4.000 4.000 4.000 3.000 3.000 3.000 4.000

4.000 4.000 3.000 3.000 3.000 4.000 4.000 3.000 4.000 2.000

5.000 4.000 2.000 2.000 4.000 3.000 3.000 4.000 4.000 3.000

3.000 3.000 4.000 3.000 3.000 3.000 3.000 4.000 3.000 4.000

3.000 4.000 3.000 4.000 3.000 3.000 3.000 4.000 3.000 4.000

4.000 5.000 5.000 5.000 5.000 4.000 4.000 4.000 4.000 3.000

4.000 4.000 4.000 4.000 4.000 4.000 3.000 4.000 4.000 4.000

4.000 4.000 4.000 4.000 4.000 5.000 5.000 5.000 5.000 5.000

5.000 5.000 5.000 3.000 5.000 5.000 5.000 5.000 5.000 5.000

5.000 5.000 5.000 5.000 5.000 5.000 5.000 5.000 5.000 5.000

5.000 5.000 5.000 4.000 4.000 5.000 4.000 4.000 4.000 4.000

5.000 5.000 5.000 5.000 5.000 5.000 5.000 5.000 5.000 5.000

5.000 5.000 5.000 5.000 5.000 3.000 4.000 4.000 4.000 4.000

4.000 4.000 4.000 3.000 4.000 4.000 4.000 4.000 4.000 4.000

4.000 5.000 4.000 5.000 4.000 4.000 4.000 4.000 4.000 5.000

4.000 5.000 5.000 5.000 5.000 5.000 4.000 5.000 4.000 4.000

5.000 5.000 5.000 5.000 5.000 4.000 4.000 4.000 4.000 4.000

5.000 5.000 5.000 4.000 5.000 5.000 5.000 5.000 5.000 4.000

5.000 5.000 5.000 5.000 4.000 5.000 5.000 5.000 5.000 5.000

5.000 5.000 4.000 4.000 4.000 5.000 5.000 5.000 5.000 5.000

4.000 4.000 4.000 4.000 5.000 5.000 5.000 5.000 5.000 5.000

5.000 4.000 4.000 4.000 4.000 4.000 4.000 4.000 4.000 5.000

5.000 5.000 5.000 5.000 5.000 3.000 3.000 3.000 3.000 3.000

2.000 4.000 2.000 3.000 2.000 3.000 3.000 2.000 3.000 3.000

2.000 2.000 2.000 2.000 3.000 2.000 3.000 3.000 3.000 2.000

4.000 4.000 4.000 4.000 4.000 3.000 4.000 5.000 3.000 2.000

4.000 4.000 4.000 3.000 4.000 5.000 3.000 2.000 4.000 4.000

3.000 3.000 4.000 3.000 4.000

RECORD NUMBER 5:

4.000 4.000 5.000 5.000 4.000 4.000 5.000 4.000 4.000 5.000

4.000 5.000 4.000 5.000 5.000 5.000 4.000 4.000 4.000 4.000

5.000 5.000 4.000 5.000 5.000 4.000 4.000 4.000 4.000 4.000

4.000 4.000 4.000 4.000 4.000 4.000 4.000 4.000 4.000 3.000

5.000 3.000 5.000 4.000 3.000 3.000 4.000 4.000 4.000 5.000

4.000 5.000 5.000 5.000 5.000 5.000 5.000 5.000 5.000 5.000

5.000 5.000 5.000 5.000 5.000 5.000 5.000 5.000 5.000 5.000

5.000 5.000 5.000 5.000 5.000 4.000 5.000 5.000 5.000 5.000

5.000 5.000 5.000 4.000 4.000 4.000 4.000 5.000 5.000 5.000

5.000 5.000 5.000 3.000 3.000 3.000 2.000 2.000 5.000 5.000

4.000 5.000 4.000 5.000 5.000 5.000 5.000 5.000 5.000 5.000

5.000 5.000 5.000 5.000 5.000 5.000 5.000 5.000 5.000 5.000

5.000 5.000 5.000 5.000 5.000 5.000 4.000 4.000 5.000 4.000

5.000 5.000 5.000 5.000 5.000 5.000 5.000 5.000 4.000 5.000

4.000 5.000 4.000 5.000 5.000 5.000 4.000 4.000 4.000 4.000

3.000 3.000 3.000 3.000 3.000 3.000 4.000 4.000 4.000 4.000

4.000 3.000 3.000 4.000 4.000 4.000 4.000 5.000 5.000 5.000

5.000 5.000 5.000 5.000 5.000 4.000 4.000 4.000 4.000 4.000

4.000 4.000 3.000 4.000 3.000 4.000 4.000 4.000 4.000 4.000

4.000 4.000 4.000 3.000 3.000 3.000 4.000 4.000 3.000 4.000

4.000 4.000 5.000 4.000 4.000 4.000 4.000 4.000 5.000 4.000

5.000 4.000 5.000 4.000 5.000 5.000 5.000 4.000 4.000 3.000

4.000 4.000 4.000 3.000 4.000 5.000 4.000 4.000 4.000 4.000

4.000 4.000 4.000 4.000 5.000 4.000 5.000 5.000 5.000 5.000

5.000 5.000 3.000 5.000 5.000 5.000 4.000 4.000 3.000 3.000

3.000 2.000 1.000 1.000 2.000 3.000 1.000 2.000 2.000 2.000

1.000 3.000 3.000 1.000 2.000 1.000 2.000 2.000 5.000 5.000

4.000 4.000 5.000 5.000 4.000 4.000 5.000 4.000 4.000 4.000

4.000 4.000 4.000 5.000 5.000 4.000 4.000 4.000 4.000 5.000

4.000 4.000 4.000 4.000 4.000 4.000 5.000 4.000 5.000 5.000

3.000 4.000 4.000 4.000 3.000 4.000 4.000 4.000 3.000 4.000

3.000 4.000 3.000 3.000 3.000 3.000 2.000 3.000 3.000 3.000

3.000 3.000 3.000 4.000 3.000 4.000 5.000 5.000 5.000 5.000

5.000 5.000 4.000 5.000 4.000 4.000 4.000 5.000 5.000 5.000

4.000 4.000 4.000 4.000 5.000 5.000 5.000 5.000 5.000 4.000

3.000 3.000 3.000 3.000 3.000 3.000 3.000 3.000 3.000 3.000

3.000 3.000 3.000 3.000 3.000 1.000 3.000 3.000 3.000 3.000

3.000 3.000 3.000 3.000 3.000 3.000 4.000 3.000 4.000 3.000

4.000 3.000 4.000 3.000 4.000 3.000 4.000 3.000 3.000 4.000

3.000 3.000 2.000 3.000 1.000 3.000 3.000 3.000 3.000 3.000

5.000 4.000 4.000 4.000 4.000 4.000 4.000 4.000 4.000 3.000

4.000 5.000 5.000 5.000 4.000 4.000 3.000 4.000 4.000 4.000

4.000 4.000 4.000 4.000 3.000 5.000 5.000 4.000 4.000 4.000

5.000 5.000 4.000 5.000 3.000 4.000 5.000 4.000 5.000 4.000

5.000 5.000 5.000 4.000 5.000 5.000 5.000 4.000 4.000 5.000

2.000 3.000 3.000 3.000 5.000 4.000 4.000 4.000 4.000 4.000

4.000 4.000 4.000 4.000 4.000 4.000 4.000 4.000 4.000 4.000

4.000 4.000 4.000 3.000 5.000 4.000 4.000 5.000 5.000 5.000

4.000 4.000 5.000 3.000 5.000 5.000 5.000 5.000 5.000 5.000

5.000 4.000 4.000 4.000 4.000 4.000 3.000 3.000 3.000 3.000

5.000 5.000 5.000 5.000 5.000 5.000 5.000 5.000 5.000 5.000

5.000 5.000 5.000 5.000 5.000 5.000 5.000 5.000 5.000 4.000

5.000 5.000 5.000 5.000 5.000 4.000 3.000 4.000 4.000 4.000

4.000 3.000 4.000 4.000 3.000 4.000 4.000 4.000 4.000 4.000

4.000 4.000 4.000 4.000 3.000 4.000 4.000 4.000 4.000 4.000

5.000 5.000 5.000 4.000 4.000 3.000 3.000 4.000 4.000 4.000

5.000 5.000 5.000 5.000 5.000 5.000 4.000 3.000 3.000 3.000

5.000 5.000 5.000 5.000 5.000 5.000 5.000 3.000 4.000 5.000

5.000 4.000 5.000 5.000 5.000 3.000 4.000 4.000 4.000 4.000

5.000 1.000 5.000 4.000 5.000 4.000 5.000 3.000 3.000 5.000

5.000 5.000 5.000 3.000 3.000 4.000 4.000 5.000 5.000 3.000

4.000 5.000 5.000 5.000 4.000 5.000 3.000 3.000 5.000 3.000

4.000 3.000 4.000 4.000 5.000 4.000 5.000 5.000 3.000 5.000

4.000 3.000 5.000 4.000 3.000 4.000 5.000 4.000 5.000 4.000

5.000 4.000 2.000 3.000 4.000 5.000 4.000 3.000 4.000 5.000

4.000 4.000 4.000 3.000 3.000 2.000 3.000 3.000 3.000 3.000

3.000 2.000 3.000 3.000 3.000 3.000 2.000 3.000 3.000 2.000

3.000 2.000 3.000 3.000 3.000 3.000 3.000 4.000 4.000 4.000

3.000 3.000 3.000 3.000 3.000 3.000 4.000 3.000 3.000 4.000

4.000 4.000 4.000 4.000 3.000 3.000 3.000 4.000 4.000 3.000

5.000 5.000 5.000 5.000 5.000 5.000 5.000 5.000 5.000 5.000

5.000 4.000 4.000 5.000 3.000 4.000 4.000 5.000 4.000 5.000

4.000 4.000 5.000 3.000 5.000 4.000 4.000 5.000 5.000 4.000

5.000 5.000 5.000 5.000 4.000 5.000 4.000 5.000 4.000 5.000

4.000 4.000 5.000 5.000 5.000 5.000 5.000 5.000 5.000 4.000

5.000 4.000 4.000 4.000 3.000 3.000 3.000 4.000 4.000 4.000

4.000 4.000 5.000 5.000 5.000 5.000 4.000 4.000 3.000 3.000

3.000 4.000 4.000 5.000 5.000 5.000 4.000 4.000 4.000 4.000

4.000 4.000 4.000 4.000 4.000 4.000 4.000 3.000 3.000 3.000

5.000 5.000 5.000 4.000 4.000 3.000 3.000 3.000 3.000 3.000

4.000 4.000 4.000 5.000 4.000 4.000 4.000 5.000 4.000 5.000

4.000 4.000 4.000 3.000 5.000 4.000 3.000 3.000 3.000 3.000

4.000 2.000 4.000 3.000 4.000 5.000 5.000 4.000 4.000 4.000

5.000 5.000 3.000 3.000 3.000 4.000 4.000 5.000 5.000 5.000

3.000 4.000 5.000 5.000 4.000 4.000 3.000 3.000 3.000 3.000

3.000 4.000 4.000 4.000 4.000 4.000 4.000 4.000 4.000 4.000

4.000 3.000 3.000 4.000 3.000 4.000 3.000 3.000 4.000 4.000

4.000 4.000 4.000 4.000 4.000 4.000 4.000 4.000 4.000 4.000

4.000 4.000 4.000 4.000 4.000 4.000 4.000 4.000 4.000 4.000

4.000 4.000 4.000 4.000 4.000 4.000 4.000 4.000 4.000 4.000

3.000 4.000 4.000 4.000 4.000 4.000 4.000 4.000 4.000 4.000

4.000 4.000 4.000 4.000 4.000 4.000 4.000 3.000 3.000 4.000

4.000 4.000 4.000 4.000 4.000 3.000 3.000 3.000 3.000 3.000

3.000 3.000 3.000 3.000 3.000 4.000 4.000 4.000 3.000 3.000

3.000 3.000 3.000 3.000 3.000 3.000 3.000 3.000 3.000 3.000

5.000 5.000 4.000 5.000 4.000 5.000 4.000 5.000 4.000 4.000

5.000 5.000 5.000 5.000 5.000 5.000 5.000 5.000 4.000 4.000

3.000 5.000 5.000 5.000 5.000 5.000 5.000 5.000 5.000 4.000

5.000 4.000 5.000 5.000 5.000 4.000 4.000 5.000 4.000 4.000

5.000 5.000 5.000 5.000 4.000 5.000 5.000 5.000 4.000 5.000

5.000 5.000 5.000 5.000 5.000 5.000 5.000 4.000 4.000 5.000

4.000 4.000 5.000 4.000 5.000 5.000 5.000 5.000 4.000 5.000

5.000 5.000 4.000 5.000 5.000 4.000 5.000 5.000 5.000 5.000

5.000 5.000 5.000 4.000 4.000 5.000 5.000 5.000 5.000 5.000

5.000 5.000 5.000 5.000 4.000 4.000 5.000 5.000 5.000 5.000

4.000 4.000 4.000 5.000 4.000 4.000 5.000 4.000 4.000 4.000

5.000 5.000 5.000 4.000 4.000 4.000 4.000 4.000 5.000 5.000

5.000 5.000 5.000 4.000 4.000 5.000 5.000 5.000 4.000 5.000

5.000 5.000 5.000 5.000 4.000 4.000 4.000 4.000 5.000 5.000

5.000 5.000 5.000 5.000 4.000 4.000 4.000 4.000 4.000 4.000

4.000 4.000 4.000 5.000 5.000 4.000 5.000 5.000 4.000 5.000

5.000 5.000 5.000 5.000 5.000 5.000 4.000 5.000 5.000 4.000

5.000 5.000 3.000 3.000 3.000 3.000 4.000 4.000 5.000 5.000

4.000 5.000 5.000 5.000 5.000 5.000 5.000 4.000 4.000 4.000

4.000 4.000 4.000 4.000 4.000 5.000 5.000 5.000 5.000 5.000

4.000 4.000 4.000 4.000 3.000 4.000 4.000 4.000 4.000 4.000

4.000 4.000 5.000 4.000 4.000 4.000 4.000 4.000 4.000 4.000

4.000 4.000 3.000 2.000 3.000 3.000 4.000 4.000 3.000 5.000

5.000 5.000 5.000 5.000 4.000 4.000 5.000 4.000 5.000 5.000

5.000 4.000 4.000 4.000 4.000 4.000 4.000 4.000 5.000 5.000

4.000 4.000 3.000 4.000 4.000 4.000 4.000 3.000 5.000 4.000

4.000 4.000 4.000 4.000 4.000 4.000 4.000 4.000 4.000 5.000

4.000 4.000 4.000 4.000 4.000 4.000 4.000 5.000 3.000 4.000

3.000 5.000 5.000 3.000 5.000 5.000 5.000 5.000 5.000 4.000

4.000 2.000 2.000 4.000 2.000 4.000 4.000 3.000 3.000 3.000

4.000 5.000 5.000 4.000 4.000 5.000 4.000 5.000 5.000 2.000

4.000 4.000 4.000 5.000 5.000 5.000 3.000 3.000 4.000 3.000

4.000 4.000 4.000 5.000 5.000 4.000 4.000 4.000 4.000 4.000

4.000 4.000 4.000 4.000 4.000 4.000 4.000 4.000 4.000 4.000

4.000 4.000 4.000 4.000 3.000 3.000 3.000 3.000 3.000 3.000

5.000 5.000 4.000 4.000 5.000 5.000 5.000 5.000 5.000 5.000

5.000 4.000 5.000 5.000 5.000 4.000 5.000 5.000 4.000 4.000

4.000 4.000 4.000 4.000 4.000 5.000 5.000 5.000 4.000 5.000

5.000 4.000 5.000 5.000 5.000 4.000 4.000 4.000 3.000 4.000

4.000 4.000 3.000 4.000 4.000 4.000 4.000 4.000 4.000 4.000

3.000 3.000 3.000 3.000 3.000 3.000 3.000 3.000 4.000 3.000

4.000 5.000 5.000 4.000 4.000 4.000 4.000 4.000 4.000 4.000

4.000 4.000 3.000 3.000 3.000 4.000 4.000 4.000 4.000 3.000

4.000 4.000 4.000 3.000 3.000 3.000 4.000 4.000 4.000 4.000

4.000 3.000 4.000 4.000 4.000 4.000 4.000 4.000 4.000 4.000

5.000 5.000 4.000 5.000 4.000 5.000 5.000 5.000 5.000 4.000

4.000 4.000 5.000 5.000 5.000 5.000 5.000 4.000 5.000 5.000

4.000 4.000 3.000 4.000 4.000 5.000 5.000 5.000 5.000 5.000

4.000 4.000 5.000 5.000 5.000 5.000 4.000 4.000 4.000 5.000

4.000 5.000 4.000 5.000 4.000 5.000 4.000 5.000 4.000 4.000

4.000 3.000 4.000 5.000 5.000 4.000 4.000 3.000 4.000 4.000

3.000 4.000 4.000 3.000 4.000 4.000 4.000 4.000 3.000 4.000

4.000 4.000 3.000 4.000 4.000 4.000 4.000 4.000 4.000 4.000

4.000 4.000 4.000 4.000 3.000 4.000 3.000 5.000 4.000 4.000

3.000 4.000 4.000 5.000 4.000 3.000 3.000 4.000 4.000 4.000

MEANS FOR MAIN EFFECTS FOR RUN 1

(s:t)xi Design

Means for t

3.860 4.200 3.909 4.139 4.109 3.773 4.120 3.750 4.063 4.207

4.247 3.624 4.267 4.186 4.014 4.472 2.760 4.353 4.187

Means for s:t

3.560 4.280 3.560 3.840 3.960 3.560 2.960 3.520 4.520 4.080

4.400 3.440 4.200 3.840 3.640 4.640 4.120 4.760 2.560 3.040

4.000 4.440 4.960 3.560 4.680 4.560 3.760 4.240 4.440 4.000

4.800 4.200 4.920 4.280 4.080 4.400 4.320 4.400 4.400 3.480

4.160 3.760 4.200 3.400 4.000 4.040 3.960 4.120 4.560 3.360

3.160 3.520 3.480 4.040 3.960 3.120 4.400 3.720 3.800 4.160

4.840 4.800 3.720 3.640 3.920 4.080 4.200 3.400 4.480 4.000

3.200 4.040 4.120 4.320 3.480 4.360 4.000 4.680 3.880 3.960

3.680 4.720 4.480 4.160 4.720 4.440 4.880 4.400 4.440 2.880

3.960 3.960 3.440 3.360 4.080 4.920 4.760 4.040 4.600 4.800

4.520 2.640 3.640 4.480 3.960 4.960 4.320 4.920 4.600 4.000

3.760 4.200 4.320 2.640 4.280 3.320 4.600 2.920 3.160 4.040

4.520 3.840 4.240 4.960 3.840 4.360 4.200 4.160 4.080 2.920

3.440 4.560 4.640 4.040 3.840 3.840 4.040 3.760 4.000 3.880

3.120 4.640 4.680 4.720 4.800 4.400 4.560 4.480 4.480 3.840

4.360 4.000 3.840 4.200 3.760 4.560 4.240 3.600 3.800 4.520

4.560 3.840 3.880 3.080 1.880 3.800 3.680 3.720 3.880 3.920

3.760 4.000 3.640 4.240 2.720 2.720 4.400 4.680 4.720 4.120

3.960 4.760 4.080 4.800 4.520 4.040 4.360 4.040 3.280 3.480

4.360 4.360 4.600 4.760 2.920 3.600 4.120 3.920 4.200 4.120

4.000 4.440 3.360 4.320 4.920 4.200 4.200 3.080 4.320 2.920

3.920 4.720 3.920 4.120 4.240 3.520 3.040 3.040 2.920 3.360

3.760 4.120 3.600 3.960 3.760 4.240 3.840 3.720 3.400 3.760

3.760 3.000 4.840 4.680 4.120 3.080 3.840 3.400 3.360 4.720

4.760 4.800 4.480 2.720 3.840 3.960 4.440 3.840 3.440 4.000

4.040 4.320 4.480 3.800 4.200 4.440 3.880 1.720 4.160 4.040

4.560 4.440 4.080 4.320 4.600 3.760 4.040 4.040 4.360 4.560

4.760 4.560 4.040 3.520 4.000 4.080 4.360 3.840 4.520 4.360

4.120 4.880 4.400 4.400 4.320 4.320 4.080 3.720 4.560 4.360

4.440 4.320 4.520 4.360 4.560 4.400 4.840 4.720 4.400 3.960

4.040 4.880 2.920 3.280 4.400 2.840 4.760 4.200 4.280 4.400

2.960 3.920 3.520 3.560 4.680 4.000 3.320 3.760 4.240 3.760

3.920 3.280 3.360 3.560 3.280 2.600 4.080 4.520 3.720 3.720

3.760 4.080 3.800 3.480 4.080 3.840 4.440 4.080 4.040 3.840

4.160 3.800 3.520 3.080 3.440 3.800 3.640 3.960 3.640 3.600

3.000 3.760 3.400 2.840 3.400 3.600 4.080 3.720 3.520 3.160

3.680 3.800 3.800 3.760 3.880 4.120 2.720 4.200 3.800 2.080

3.480 2.880 3.360 2.040 3.720 4.760 4.240 4.920 4.080 4.880

3.800 4.720 4.800 3.800 3.760 3.960 4.640 4.080 4.160 3.720

3.760 3.960 4.680 4.160 4.600 3.600 4.560 4.440 4.480 4.240

4.680 4.680 3.800 4.560 2.920 4.960 3.480 3.360 3.680 3.880

4.000 4.960 4.200 4.000 4.880 4.440 4.360 4.640 4.800 4.480

3.880 4.040 4.240 3.960 3.840 4.560 4.240 4.560 3.840 4.000

3.800 3.640 4.160 3.680 4.320 4.080 3.720 4.920 4.280 4.520

4.480 3.800 2.880 4.160 4.440 3.760 3.240 4.680 4.440 4.520

4.040 4.400 4.640 4.400 4.680 4.840 4.760 4.120 4.360 4.640

4.680 4.040 4.760 4.040 4.360 4.480 4.480 4.560 4.000 3.520

4.200 2.960 4.400 2.760 1.440 3.640 1.600 2.200 3.400 1.600

1.920 3.480 2.480 2.560 1.600 3.560 3.560 1.840 1.240 4.280

4.360 3.680 4.800 4.360 4.640 4.480 4.800 3.560 4.120 4.480

4.160 4.840 4.440 4.600 3.760 4.720 4.320 4.280 4.280 4.240

4.560 4.400 3.280 4.120 3.440 4.160 4.680 3.800 4.840 4.680

4.760 4.400 3.800 4.200 4.440 3.680 4.200 4.760 3.400 4.520

Means for i

4.126 4.121 4.138 4.130 4.023 3.998 4.023 4.011 3.968 3.751

4.132 4.206 4.234 4.306 4.098 3.964 3.896 3.892 3.960 3.638

3.853 3.974 3.755 3.655 3.985

ANOVA TABLE FOR RUN 1

(s:t)xi Design

-----------------------------------------------------------------------------

Effect df T SS MS VC

-----------------------------------------------------------------------------

t 18 212818.41265 1513.84140 84.10230 0.11059

s:t 511 216482.68000 3664.26735 7.17078 0.26909

i 24 211678.51132 373.94008 15.58084 0.02702

ti 432 213673.73085 481.37813 1.11430 0.02432

si:t 12264 222777.00000 5439.00180 0.44349 0.44349

-----------------------------------------------------------------------------

Mean 211304.57125

-----------------------------------------------------------------------------

Total 13249 11472.42875

-----------------------------------------------------------------------------

Grand Mean: 3.99343

EXPECTED MEAN SQUARE EQUATIONS FOR RUN 1

(s:t)xi Design

EMS(t) = 1.000*VC(si:t) + 27.584*VC(ti) + 25.000*VC(s:t) + 689.602*VC(t)

EMS(s:t) = 1.000*VC(si:t) + 25.000*VC(s:t)

EMS(i) = 1.000*VC(si:t) + 33.487*VC(ti) + 530.000*VC(i)

EMS(ti) = 1.000*VC(si:t) + 27.584*VC(ti)

EMS(si:t) = 1.000*VC(si:t)

*** EMS matrix is upper diagonal***

Date and time at beginning of Run 1: Sun Jan 8 16:34:59 2017

Processor time for run: 1 seconds
